# A Developmental Switch in Place Cell Accuracy Coincides with Grid Cell Maturation

**DOI:** 10.1016/j.neuron.2015.05.011

**Published:** 2015-06-03

**Authors:** Laurenz Muessig, Jonas Hauser, Thomas Joseph Wills, Francesca Cacucci

**Affiliations:** 1 Department of Neuroscience, Physiology, and Pharmacology, University College London, Gower Street, London WC1E 6BT, UK; 2 Department of Cell and Developmental Biology, University College London, Gower Street, London WC1E 6BT, UK

## Abstract

Place cell firing relies on information about self-motion and the external environment, which may be conveyed by grid and border cells, respectively. Here, we investigate the possible contributions of these cell types to place cell firing, taking advantage of a developmental time window during which stable border cell, but not grid cell, inputs are available. We find that before weaning, the place cell representation of space is denser, more stable, and more accurate close to environmental boundaries. Boundary-responsive neurons such as border cells may, therefore, contribute to stable and accurate place fields in pre-weanling rats. By contrast, place cells become equally stable and accurate throughout the environment after weaning and in adulthood. This developmental switch in place cell accuracy coincides with the emergence of the grid cell network in the entorhinal cortex, raising the possibility that grid cells contribute to stable place fields when an organism is far from environmental boundaries.

## Highlights

• During early development, place cell maps are maximally stable near boundaries
• At weaning age, place cell maps switch to become equally accurate throughout space
• This developmental switch coincides with the emergence of the grid cell network
• Boundary cells may support place maps at edges, and grid cells in the environment center

## Teaser

Muessig et al. show that during early post-natal development, hippocampal place cell maps are maximally accurate near environmental boundaries. Coinciding with grid cell emergence, place maps become accurate throughout space, suggesting that grid cells may stabilize place maps far from boundaries.

## Introduction

Place cells are pyramidal cells in the CA1 and CA3 fields of the hippocampus that fire only when an animal visits selective regions of the environment (“place fields”). Collectively, their firing is thought to constitute a “cognitive map” of an environment, allowing an animal to locate itself and navigate to a goal (O’Keefe and Nadel, 1978).

Place cell firing is thought to integrate inputs from several other types of spatially tuned neurons (Zhang et al., 2013). These include border cells (Solstad et al., 2008), which fire close to the boundaries of an environment, and grid cells (Hafting et al., 2005), which fire in a regular, hexagonally symmetric series of locations across the whole environment; both are found in the medial entorhinal cortex (mEC). Grid cells are thought to encode an intrinsic metric for space based on self-motion information (Burak and Fiete, 2009; Burgess et al., 2007; Fuhs and Touretzky, 2006; Hafting et al., 2005; McNaughton et al., 2006; Zilli and Hasselmo, 2010), whereas boundary-responsive cells such as border cells may, instead, allow external sensory information to stabilize grid and place cell maps near the boundaries of the environment (Burgess et al., 2007; Hartley et al., 2000; Lever et al., 2009; Savelli et al., 2008; Solstad et al., 2008).

Following the discovery of grid cells in the mEC, several theoretical models put forward the hypothesis that place cell firing could be derived solely from grid cell inputs (Fuhs and Touretzky, 2006; Monaco and Abbott, 2011; O’Keefe and Burgess, 2005; Solstad et al., 2006). However, more recent evidence has shown that place fields can exist in the absence of regular grid cell firing both during post-natal development (Langston et al., 2010; Wills et al., 2010) and in adulthood (Koenig et al., 2011). This leaves open the question of the exact contribution of grid cell input to place cell firing.

In this study, we use a developmental model to address this unresolved question. We take advantage of the fact that, during the post-natal development of the hippocampal formation, the first adult-like grid cells emerge at around weaning age (Post-natal day 21 [P21]; Wills et al., 2010), whereas hippocampal CA1 pyramidal cells show spatially tuned and stable firing at least four days earlier, at P16 (Langston et al., 2010; Wills et al., 2010). This developmental timeline provides an opportunity to study the nature of place cell firing before the onset of stable grid cell firing.

A putative stabilizing signal to place cells before grid cells emerge are boundary-responsive cells. In particular, recent work has shown that mEC border cells emerge at P17 and may, therefore, drive stable place cell firing before weaning age (Bjerknes et al., 2014; Wills et al., 2010). We hypothesized that, in pre-weanling animals, when border cells may be the sole stabilizing input to place cells, place fields will be more numerous and more stable close to boundaries. Because of the fact that most boundary-responsive cells are narrowly tuned to locations close to environmental boundaries (Bjerknes et al., 2014; Lever et al., 2009; Solstad et al., 2008; Stewart et al., 2014), place cells should be less stable and less accurate in the center of an open field environment at this age. By contrast, the emergence of stable grid cell firing at weaning age might mark the transition to place cell firing that is stable and accurate throughout the environment.

## Results

We recorded 813 place cells from the hippocampal CA1 field in pups aged between P14 and P30 and 201 place cells from adult rats under similar conditions (see Experimental Procedures).

**Figure 1:**
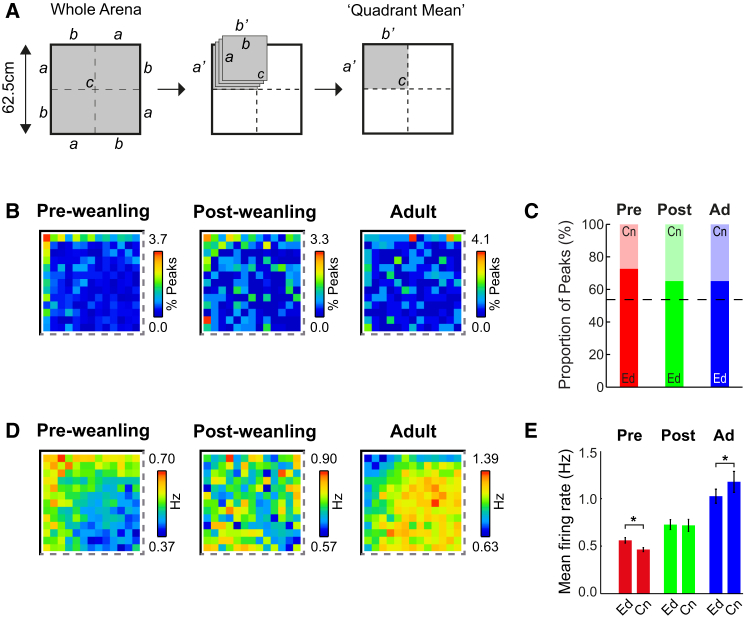
Place Cell Firing Is More Concentrated Close to Environment Boundaries in Pre-weanling (P14–P21) than in Post-weanling (P22–P30) and Adult Rats (A) Quadrant mean map construction. The full map is divided into quadrants rotated around the center of the environment (*c*) such that all walls *a* are mapped onto *a*’ and all walls *b* are mapped onto *b*’. (B) False-color quadrant mean maps of the distribution of peak firing rate locations (expressed as percent of all peaks). (C) Proportion of place cell peaks in “edge” (“Ed”, bottom part of each bar) versus “center” (Cn, top part of each bar) zones of the environment. The black dashed line indicates the expected proportion for an even distribution of peaks across the environment. Ad, adult. (D) Quadrant mean rate maps of the overall, unsmoothed firing rate (in Hz) for all recorded place cells in each age group. (E) Mean place cell firing rate (± SEM) in edge versus center zones of the environment. ^∗^p < 0.01 level.

An analysis of the positions of place cell firing fields in the recording arena reveals that there is a greater concentration of place fields close to boundaries in pre-weanling pups (P14–P21) compared with post-weanling (P22–P30) or adult rats (Figure 1B; maps are shown in “quadrant mean” format, Figure 1A). To quantify this phenomenon, we calculated the proportion of place cell peaks in two zones of the environment: “edge” and “center” (≤ and >10 cm from the nearest wall, respectively; Figure 1C). All age groups show more place fields in the “edge” zone than expected from an even distribution (e.g., one-sample Z test versus the expected proportion for even distribution; for adults, Z = 3.2, p = 0.001). However, pre-weanling animals show a higher proportion of place fields in the “edge” zone compared with post-weanling or adult rats (Figure 1C; χ^2^ test versus equal proportion in all age groups; χ^2^(2) = 6.53, p = 0.038; two-sample Z test, pre versus post, Z = 2.04, p = 0.04; two-sample Z test, pre versus adult, Z = 2.04, p = 0.04).

Because many place cells in pre-weanling rats have multiple discrete place fields, we also constructed the mean rate maps of all recorded cells to give a fuller picture of place field location. Place cell firing is concentrated toward the boundaries in pre-weanling animals and toward the environment center in adults, whereas no bias exists in post-weanling rats (Figure 1D). When comparing mean firing rates in the two zones of the environment, we find that firing rates are higher in the “edge” in pre-weanling pups and lower in adults (Figure 1E; ANOVA zone^∗^age F_2,1011_ = 8.8, p < 0.001; post hoc comparison within age group (simple main effects [SMEs]), SME zone_(pre-wean)_, p = 0.001; SME zone_(adult)_, p = 0.004), with no differences in post-weanling animals (SME zone_(post-wean)_, p = 0.88). The developmental trends in place cell field position and firing distribution are also visible in individual animals (Figures S1A and S1B; Supplemental Experimental Procedures) and occur abruptly between P20-21 and P22-23 (Figures S1F–S1I), suggesting that a step change in the distribution of the hippocampal representation of space occurs around weaning age. In pre-weanling rats, place cell firing is concentrated near boundaries, consistent with the hypothesis that, at this age, place cells receive spatial input from border cells.

**Figure 2:**
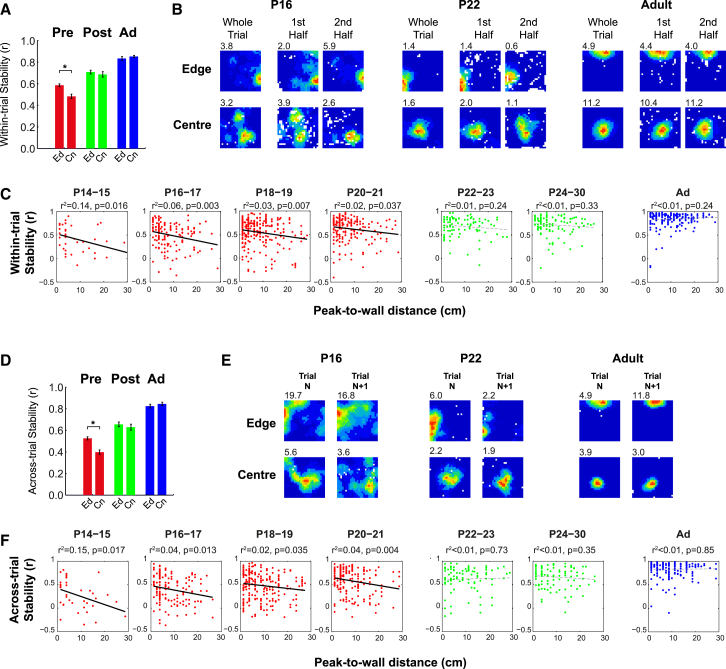
Place Fields Are More Stable Close to Environmental Walls in Pre-weanling Pups (A) Mean within-trial stability (± SEM) of place cells with peak firing locations in the edge and center zones of the environment. (B) False-color firing rate maps from representative example place cells showing within-trial stability at P16, P22, and adult. Within each age group, the maps show, from left to right, the whole recording session, the first half of the session, and the second half of the session for place fields with firing peaks located close (top) or far (bottom) from a wall (stability values for examples lie within SD of the mean of the respective population). (C) Scatterplots of within-trial stability versus distance from the peak to the nearest wall with linear regression lines of best fit. Solid black lines are significant at the p < 0.05 level, and r^2^ and p for regression are shown above the plots. (D) Mean across-trial stability (± SEM) of place fields with peak firing locations in the edge and center zones. (E) Firing rate maps showing example across-trial stability at P16, P22, and adult. Within age groups, the left and right columns show two recording sessions separated by 15 min. (F) Scatterplots of across-trial stability versus distance to wall with lines of best fit. Solid black lines are significant at the p < 0.05 level, and r^2^ and p for regression are shown above the plots.

Correspondingly, we found that, only in pre-weanling animals, place fields closer to environmental walls (“edge” zone) are significantly more stable than those located in the middle of the environment (“center” zone; Figures 2A and 2B; within-trial stability, ANOVA age^∗^zone, F_2,1005_ = 3.2, p = 0.042, SME zone_(pre-wean)_ p < 0.001; see Figure 2B for example place fields). Furthermore, the stability of place fields recorded from pre-weanling rats (but not from post-weanling or adult rats) is inversely correlated to the distance from environmental boundaries (Figure 2C). The regression line slope for all pre-weanling data is also significantly steeper than that for post-weanling data (slope constants: pre, −8.3 × 10^3^; post, −3.3 × 10^3^; t = 1.83, degrees of freedom [df] = 808, p = 0.03 [one-tailed]). In summary, during development, the hippocampal map of space is initially only stable close to environmental boundaries and becomes as stable away from these boundaries from weaning age onward.

We tested whether the inhomogeneity in within-trial stability would also apply to across-trial stability. We found that place fields near walls have greater across-trial stability in pre-weanling pups but not in post-weanling or adult animals (Figures 2D and 2E; ANOVA age^∗^zone, F_2,943_ = 5.6, p = 0.004; SME zone_(pre-wean)_ p < 0.001; SME zone_(Post-wean);_ p = 0.46; SME zone_(Adult)_, p = 0.76; see Figure 2E for example place fields). There is a significant inverse relationship between place field stability and distance to wall for all 2-day age groups between P14 and P21, but not for P22 and older (Figure 2F), and the regression slope for all pre-weanling data is significantly steeper than that of post-weanling data (slope constants: pre, −8.6 × 10^3^; post, −0.9 × 10^3^; t = 2.85, df = 767, p = 0.004 [one-tailed]). In conclusion, the pattern of place field stability between different visits to the same environment recapitulates that of within-trial stability. Before weaning, stability is lower further from boundaries, and, after weaning, stability is equal throughout the environment.

The switch between place maps that are selectively more stable close to walls (both within- and across-trial) to ones that are equally stable throughout the environment is also apparent in individual animals (Figures S1C–S1E) and occurs abruptly between P20-21 and P22-23 (Figures S1J–S1O; Figures 2C and 2F). Furthermore, these developmental changes are independent of other behavioral and physiological changes occurring during the same developmental period (Figure S2; with a single exception, Figure S2R).

**Figure 3:**
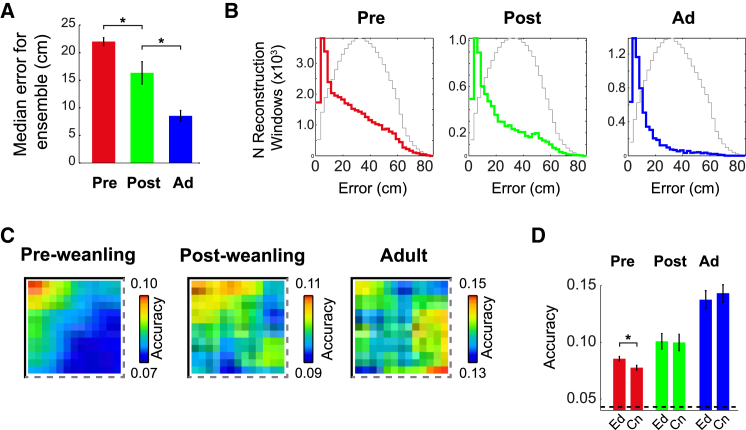
In Pre-weanling Pups, the Accuracy of Position Decoding Is Higher near Environment Boundaries (A) Median reconstruction error per ensemble for each age group (mean ± SEM). ^∗^p < 0.01 level. (B) Distribution of errors for all 1-s reconstruction time windows in each age group. Colored lines show error distributions for real data, and gray lines show errors from spatially shuffled data (Experimental Procedures). (C) Quadrant mean false-color heat map of reconstruction accuracy (1 / (error + 1)) for each age group. (D) Mean accuracy (± SEM) in “edge” and “center” zones of the environment. The black dashed line indicates the mean expected accuracy from decoding spatially shuffled data (Experimental Procedures). ^∗^p < 0.01 level.

To investigate whether the observed differences in place cell firing near and far from boundaries in pre-weanling rats affect the ability of the hippocampus to accurately encode position, we tested whether a Bayesian decoding algorithm (Zhang et al., 1998; Experimental Procedures) could reconstruct the rat’s location more accurately close to boundaries in pre-weanling rats from the firing of all recorded CA1 pyramidal cells. First, we established that Bayesian decoding can reconstruct position in developing rats. As expected, the overall reconstruction error is higher in developing than in adult rats (ANOVA age F_2,76_ = 31, p < 0.001; Tukey honestly significant difference [HSD]; all groups different at p < 0.01; Figure 3A), but, for both pre-and post-weanlings, the modal reconstruction error is the same as in adult rats (2.5–5cm), and the distribution of errors is significantly different from that expected from random reconstruction (Kolmogorov-Smirnov [KS] test: pre-wean, k = 0.23, p < 0.001; post-wean, k = 0.35, p < 0.001; Figure 3B; Experimental Procedures). Examining the spatial biases of decoding error, we found that reconstruction accuracy (1 / (error + 1)) is higher near boundaries in pre-weanling but not in post-weanling or adult rats (Figures 3C and 3D; ANOVA age^∗^zone, F_2,73_ = 8.5, p < 0.001; SME zone_(pre-wean)_ p < 0.001; SME zone_(post-wean)_, p = 0.91; SME zone_(Adult)_, p = 0.085). This result is independent of behavioral biases or the amount of previous experience of the environment (Figures S3A–S3D; Supplemental Experimental Procedures) and is not related to geometrical constraints on accuracy scores at the edge of the environment (Figures S3E–S3L).

In pre-weanling pups, the place cell representation of space affords less accurate self-localization in the center of the environment than near boundaries, whereas, in post-weanling and adult animals, the place cell code is evenly accurate throughout the explored space.

## Discussion

We have demonstrated an important developmental step change in the nature of the hippocampal representation of space in rats. Before weaning, the hippocampus encodes space more accurately close to boundaries (where input from border cells would be maximal; Bjerknes et al., 2014), whereas, after weaning, the accuracy of the hippocampal representation of space appears to be even throughout the environment. These findings are independent of physiological and behavioral changes taking place during development, and, therefore, represent a genuine change in hippocampal processing, taking place around weaning age.

This sharp developmental switch coincides with the sudden emergence of a stable grid cell network in the mEC. In animals tested under the same experimental conditions, grid cells first emerge at P20-21, but the proportion of grid cells is extremely low at these ages and significantly less than that observed in the adult. At P22-23, the percentage of mEC cells classified as grid cells suddenly reaches a level that is not significantly different to that observed in the adult (Wills et al., 2010; Figure S1P–S1R). Furthermore, in vitro recordings show that mEC stellate cell network synchronization significantly increases at P22 (Langston et al., 2010). This suggests that the widespread recurrent network thought to be necessary for grid cell activity (Burak and Fiete, 2009; Bush and Burgess, 2014; Fuhs and Touretzky, 2006; McNaughton et al., 2006; Zilli and Hasselmo, 2010) emerges at this age.

We interpret our results to suggest that grid cells may be necessary to provide a stable and accurate representation of position throughout an environment when the organism is far from environmental landmarks or boundaries. When place cells do not receive grid cell input, as in pre-weanling pups, error in their estimate of location increases when animals are further from boundaries. This hypothesized gain of function grid cells would provide to place cells is consistent with their widely proposed role in path integration (Burak and Fiete, 2009; Burgess et al., 2007; Fuhs and Touretzky, 2006; Hafting et al., 2005; Hasselmo et al., 2007; McNaughton et al., 2006). By calculating an estimate of position on the basis of self-motion cues, the role of grid cells may be to allow an accurate representation of position even when environmental cues are relatively sparse, for example, in darkness or in the center of an open field environment (Bush et al., 2014; Poucet et al., 2014). This interpretation is also consistent with recent evidence from adult rats undergoing medial septum inactivation (which disrupts theta sequences and grid cell firing; Brandon et al., 2011; Koenig et al., 2011; Wang et al., 2015) while exploring a large novel environment. Most CA1 cells did not exhibit spatial firing, but those that did had place fields at the edges of the environment (Wang et al., 2015).

Alternative explanations of our findings could involve intra-hippocampal mechanisms such as changes in synaptic plasticity (Blair et al., 2013). The age at which theta sequences emerge remains unknown, but the distribution of place fields in adults with disrupted theta sequences (Wang et al., 2015) might suggest this as another candidate explanation. Alternatively, it is possible that a single mechanism might underlie both the emergence of grid cells and the stabilization of place maps observed at weaning. For example, at weaning, the proportion of theta-modulated cells in CA1 and entorhinal cortex reaches adult values (Wills et al., 2010). The maturation of theta rhythmicity at weaning might underlie the stabilization of place fields into open space, either directly or indirectly by spurring the emergence of grid cells in the entorhinal cortex.

We have demonstrated that, at ages when border cells are present (Bjerknes et al., 2014) but grid cells have yet to emerge (Wills et al., 2010), place cells are more numerous and more stable close to boundaries. This developmental pattern may provide the first experimental evidence for a further hypothesis: that boundary-responsive cells such as border cells or boundary-vector cells (Hartley et al., 2000; Lever et al., 2009) “anchor” place and grid cell maps by providing a stabilizing input when an animal is close to environmental boundaries (Burgess et al., 2007; Savelli et al., 2008; Solstad et al., 2008). The concentration of place cell fields close to boundaries in pre-weanling pups is also consistent with the theory that place fields may be constructed from inputs from boundary-responsive cells (Hartley et al., 2000). Our results also suggest that boundary-responsive cells may be a foundational spatial signal (Bjerknes et al., 2014; Wills et al., 2010; F. Cacucci et al., 2013, Soc. Neurosci., abstract 485.16), along with head direction responses (Langston et al., 2010; Taube et al., 1990; Wills et al., 2010), during the ontogeny of hippocampal spatial representations.

Our results also offer a functional explanation as to why hippocampus-dependent behavior emerges around weaning age in rats: the first evidence of learning on spatial memory tasks appears at this age (Green and Stanton, 1989; Rauch and Raskin, 1984; Rudy et al., 1987; Schenk, 1985). Given the relationship between place cell firing and spatial behavior in adult rats (Lenck-Santini et al., 2002; O’Keefe and Speakman, 1987), this behavioral transition seems likely to be due to place cell maturation. However, previous studies of hippocampal development (Langston et al., 2010; Scott et al., 2011; Wills et al., 2010) have failed to find any candidate sharp changes in the functional properties of pre- and post-weanling place cells that might underlie the switch to a behaviorally functional navigation system. Our data show such a functional step change in CA1 place cells occurring precisely at weaning age, suggesting that the hippocampus supports spatial learning and memory only after the emergence of a cognitive map equally stable and accurate throughout an environment. This, in turn, may rely on the emergence of a grid cell network in the mEC.

## Experimental Procedures

### Subjects

43 male Lister Hooded rat pups, aged P12–P22 and weighing 24–64 g on the day of surgery, were used as subjects. Litters were bred in-house and remained with their dams until weaning (P21). Rats were maintained on a 12:12 hr light:dark schedule (lights off at 12:00). At P4, litters were culled to 8 pups/dam to minimize inter-litter variability. After surgery, each pup was separated from the mother for 30 min to 2 hr per day to allow for electrophysiological recordings. 13 male Lister Hooded adult rats, aged 4–6 months at the time of recording, were included in the study to provide a comparison for the pup data. Data from 17 of the subjects in this study (14 rat pups, 368/813 place cells; 3 adult rats, 43/201 place cells) have also contributed toward a previously published study (Wills et al., 2010). There were no differences in procedure between the two groups. The methods set out below apply equally to both groups of rats. All experiments were carried out in accordance with the relevant UK legislation (ASPA 1986).

### Surgery and Electrode Implantation

Rats were anesthetized using 1%–2% isoflurane and 0.15 mg/kg body weight buprenorphine. Rats were chronically implanted with microdrives loaded with 4-8 tetrodes (HM-L-coated 90% platinum/10% iridium 17-μm-diameter wire) aimed at the hippocampal CA1 region (2.9 mm posterior and 1.8 mm lateral to bregma). After surgery, rats recovered in a heated chamber (10–30 min) and were then returned to their mothers.

### Single-Unit Recording

Rats were allowed 1 day of postoperative recovery, after which electrodes were advanced by 62–250 μm/day until the CA1 pyramidal layer was identified by the presence of complex spike cells and 200-Hz “ripple” fast oscillations. At this point, recording sessions began. Single-unit data were acquired using an Axona DACQ system. Light-emitting diodes (LEDs) were used to track the position and directional heading of the animal. Isolation of single units from multi-unit data was performed manually on the basis of peak-to-trough amplitude using the software package TINT (Axona). Isolated single units were only used for further analysis if they fired ≥75 spikes in a trial.

### Classification of Single Units as Complex Spike Cells

Single units recorded in the CA1 were classified into complex spike cells (putative pyramidal cells) and putative interneurons using k-means clustering based on the following parameters: spike width (peak to trough); burst-firing at 3–10 ms, as assessed by the first moment of the temporal autocorrelogram, within a 50-ms window; and the mean firing rate of the cell (Csicsvari et al., 1999). If a cell was recorded on multiple trials, the trial with the highest mean rate was used to define these values. Because the physiological properties of CA1 neurons change during development (Wills et al., 2010), adult and pup data were clustered separately.

### Behavioral Testing

Single-unit activity was recorded while rats searched for drops of soya-based infant formula milk randomly scattered in a square, light gray wooden box (walls, 62.5 cm long and 50 cm high) placed on a black plastic platform. Trials were 10–15 min long. The fixed apparatus of the laboratory provided distal visual cues. Rats were kept in a separate holding box between recording trials (inter-trial interval, 15 min). Each rat was given between 1–4 recording trials per session. The median number of previous exposures to the recording environment was 11 (minimum = 0; maximum = 44; quartile range, 6–18).

### Construction of Firing Rate Maps

The edges of the visited environment were defined as the line of camera pixels (2.5 mm wide) furthest from the center of the environment where the total dwell time was ≥1 s. Positional data within the visited edges of the environment were then sorted into 2.5 × 2.5 cm spatial bins. Data were included in further analyses only if total path length >45 m and the rat visited ≥94% of the total surface area of the arena (≥585 of 625 total spatial bins). All spike and positional data were filtered to remove periods of immobility (speed, <2.5 cm/s for pups and <5 cm/s for adults). The total dwell time and spike count for the whole trial were then calculated for each spatial bin. The binned data were then smoothed using adaptive smoothing (Skaggs et al., 1996). In brief, to calculate the firing rate for a given bin, a circle centered on the bin was gradually expanded in radius *r* until

$$r\geq \frac{\alpha }{d\sqrt{s}},$$

where α = 200 and *d* and *s* are the dwell time (in seconds) and the number of spikes lying within the circle, respectively. The firing rate assigned to the bin was then set equal to *s/d*. The exception to this procedure consisted of the overall mean rate maps for all cells (Figures 1D and 1E); here, no smoothing was applied.

### Criteria for Classification of Place Cells

Complex spike cells were classified as place cells on the basis of the spatial information of their rate maps, expressing the extent to which a cell’s firing can be used to predict the position of the animal. The estimate of the mutual information I(R|X) between the firing rate R and location X is

$$I\left(R|X\right)\approx \sum_{i}p\left({\vec{x}}_{i}\right)f\left({\vec{x}}_{i}\right)\log_{2}\left(\frac{f\left({\vec{x}}_{i}\right)}{F}\right),$$

where $p\left({\vec{x}}_{i}\right)$ is the probability for the animal being at location ${\vec{x}}_{i}$, $f\left({\vec{x}}_{i}\right)$ is the firing rate observed at ${\vec{x}}_{i}$, and F is the overall firing rate of the cell (Skaggs et al., 1996). I(R|X) was then divided by the mean firing rate of the cell, giving an estimate in bits/spike. Cells were classified as place cells if their spatial information exceeded a threshold defined as the 95^th^ percentile of a population of spatial information scores derived from age-matched, spatially shuffled data (Wills et al., 2010).

### Quantification of Place Field Position and Stability

Place field location was defined as the position of the peak rate pixel in the rate map. Field-to-wall distance was defined as the minimum distance from the field peak to the environment edges. Across-trial stability was defined as the correlation (Pearson’s r) between spatially corresponding bins from two consecutive trials, excluding bins with a firing rate of 0 Hz in both trials. Trial pairs were used if a complex spike cell was classified as a place cell on at least one of the trials. Within-trial stability was the correlation between the spatially corresponding bins of the rate maps from the temporal first and last halves of the trial. If a place cell was recorded for more than one trial, the stability and peak-to-wall distance for that cell were defined as the mean over all trials recorded. Stability is displayed as r values; however, these were Z-transformed for ANOVAs. For analysis of peak position, to avoid the centralizing tendency of averaging peak positions, only one trial for each cell was used: that on which the cell was first defined as a place cell. The difference between stability versus distance-to-wall regression slopes was compared using a t test (Supplemental Experimental Procedures).

### Bayesian Reconstruction of Position

The rat’s position was reconstructed following (Zhang et al., 1998). For each 1-s reconstruction time window, the probability of the rat being in a spatial bin *x*, given the numbers of spikes fired an ensemble of N cells, represented by the vector ***n***, was defined as:

$$P\left(x\text{|}n\right)=\;\frac{P\left(n\text{|}x\right)\text{P}\left(x\right)}{P\left(n\right)}.$$

P(*x*) is the probability that the rat was at position *x*, defined as the ratio between the dwell time for spatial bin *x*, and the length of the trial. P(n|x), the probability of N cells in the ensemble firing n spikes in time window T, given that the rat was at position x, was derived from the firing rate map for each cell *i* as follows:

$$P\left(n\text{|}x\right)=\prod_{i=1}^{N}P\left(n_{i}\text{|}x\right)\;=\prod_{i=1}^{N}\frac{{\left(\tau f_{i}\left(x\right)\right)}^{n_{i}}}{ni!}\text{exp}\left(-\tau f_{i}\left(x\right)\right),$$

where f_i_(*x*) is the mean firing rate of cell *i* at position *x*, and τ is the length of the time window in seconds. The probability P(***n***) was determined by normalizing the conditional probability P(x|n) so that the sum of P(x|n) over x was equal to 1. The calculation of P(x|n) was made with reference to only spiking in the current time window T; i.e., the prior probability distribution on the basis of the previous reconstruction at T_t-1_ was assumed to be flat.

For every time window T, P(x|n) was calculated for every spatial bin, and the bin with the maximum P was considered to be the reconstructed position. The reconstruction error for T was then defined as the linear distance between the reconstructed position and the mean actual position of the rat during time window T. To determine whether spatial biases exist in the distribution of successful reconstructions, error values were converted to accuracy, *a*, as follows:

a=1/(e+ε),

where Ɛ is a constant (set to 1 cm) so that very small errors do not have an undue influence on overall accuracy.

The reconstruction analysis was based on the spiking of all recorded complex spike cells (putative pyramidal projection cells) to provide an estimate of the location information available to downstream brain areas. To allow sufficient data for successful reconstruction, only sessions with ≥10 complex spike cells were used. Time windows in which mean speed was below the threshold for immobility were excluded. Reconstruction errors expected from spatially shuffled data were generated by randomly reassigning the identities of the rate maps with respect to the spike trains (10,000 times) before applying the decoding algorithm.

## Supplemental Information

Document S1. Supplemental Experimental Procedures and Figures S1–S3

Document S2. Article plus Supplemental Information
