# Supplementary material for: A Developmental Switch in Place Cell Accuracy Coincides with Grid Cell Maturation
Source: Neuron. 2015 Jun 3;86(5):1167–73. doi: 10.1016/j.neuron.2015.05.011 (PMC4460188; doi:10.1016/j.neuron.2015.05.011)
Supplement: Document S1. Supplemental Experimental Procedures and Figures S1–S3 [file mmc1.pdf]

Neuron

Supplemental Information

A Developmental Switch in Place Cell Accuracy Coincides with Grid Cell Maturation

Laurenz Muessig, Jonas Hauser, Thomas Joseph Wills, and Francesca Cacucci

Figure S1 (related to Figure 1 and Figure 2)

Analysis of Individual Animals

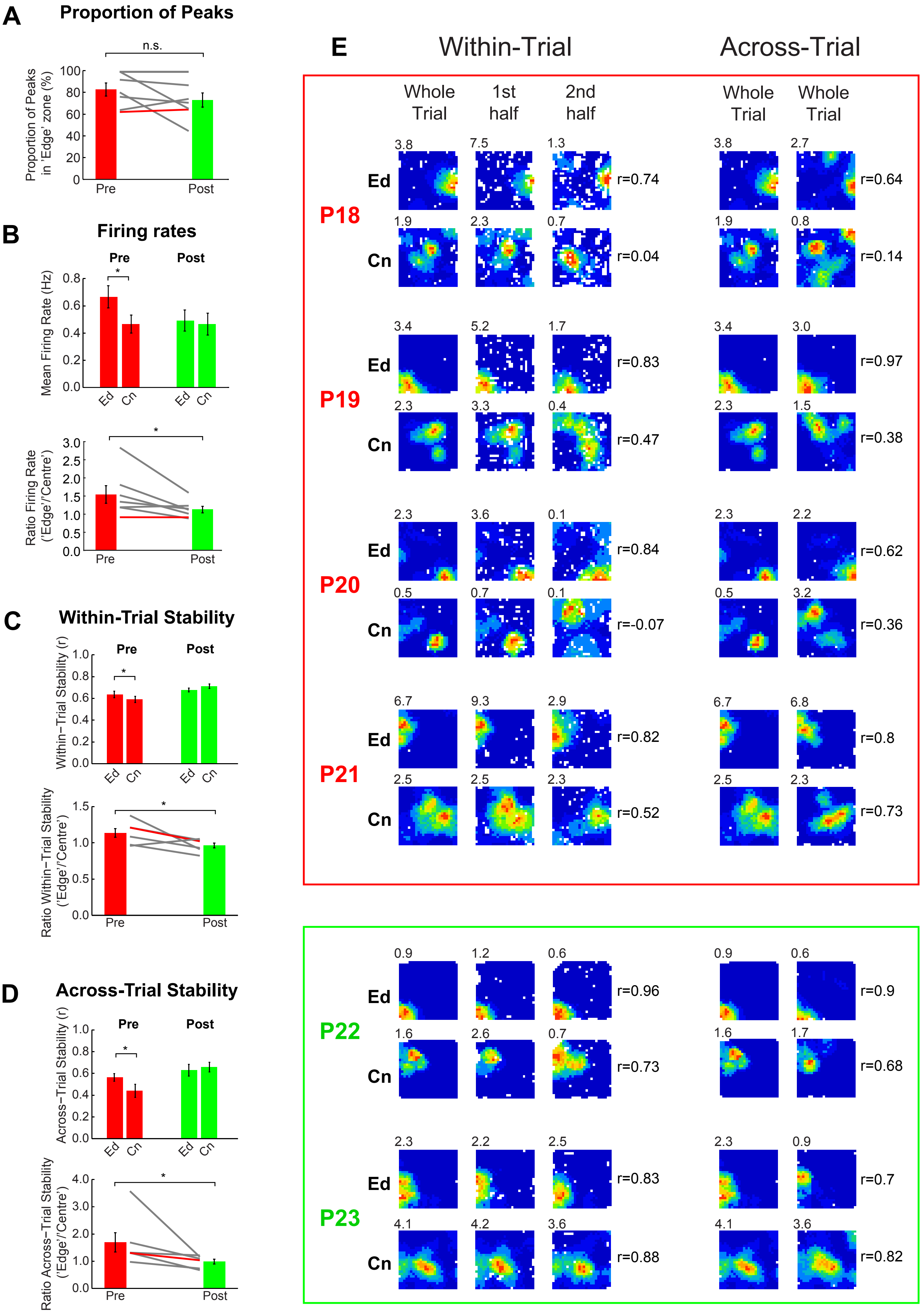

Abrupt Switch Between ‘Edge’ and ‘Centre’ Coding

Distribution of Peaks

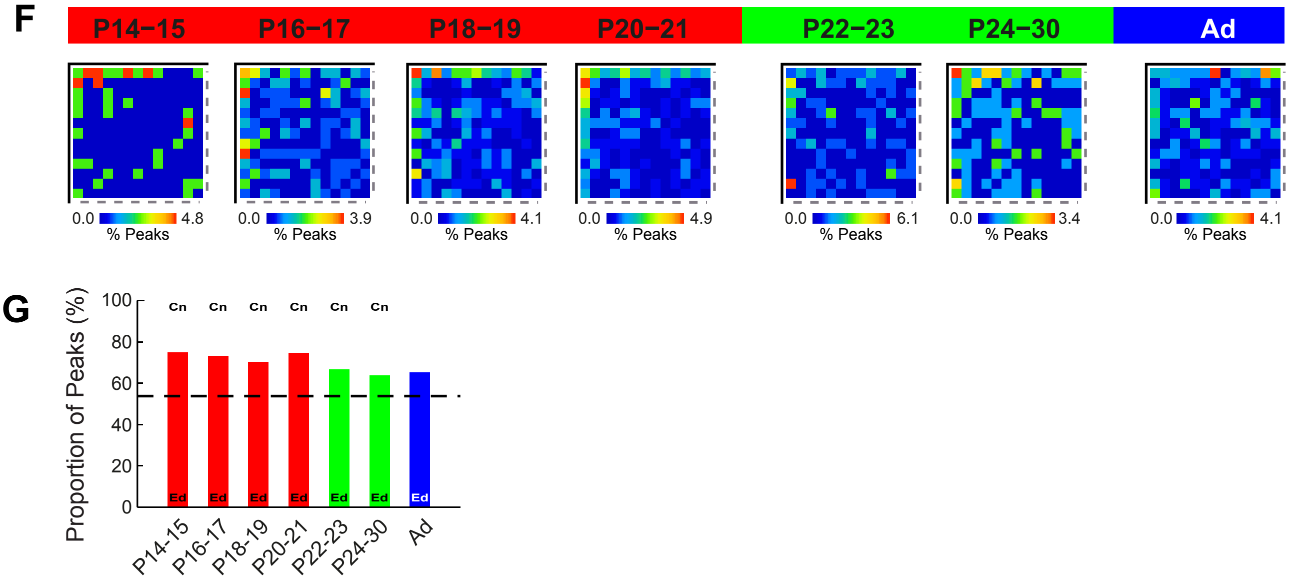

Mean Rate Maps

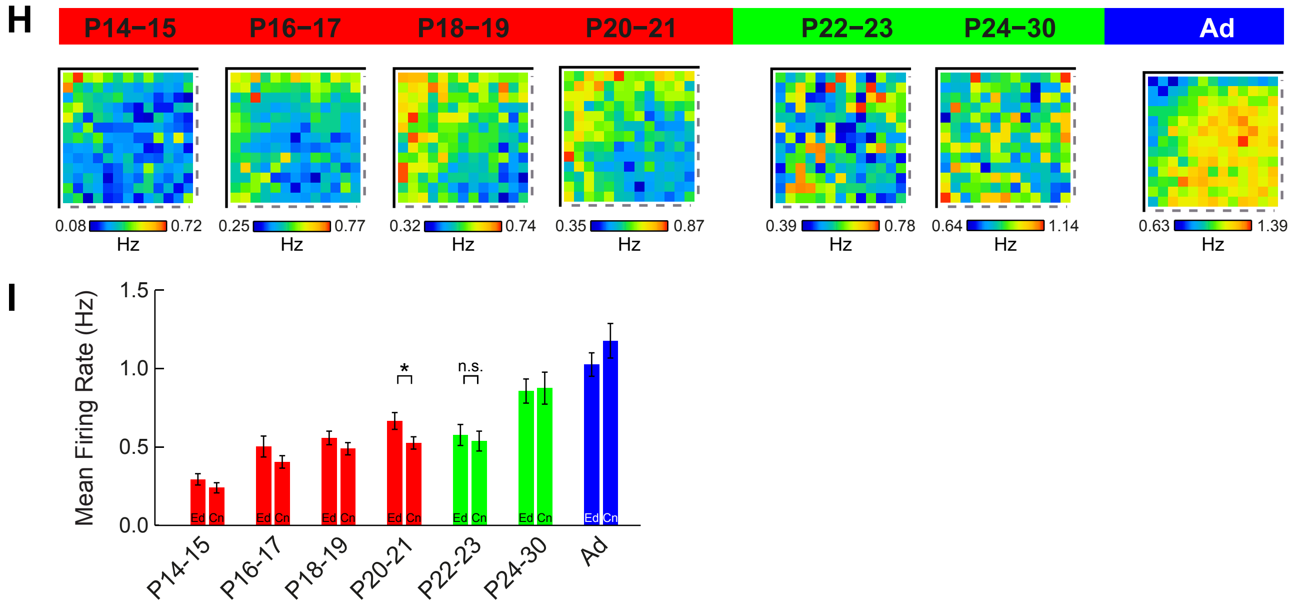

Place Cell Stability in ‘Edge’ versus ‘Centre’ zones

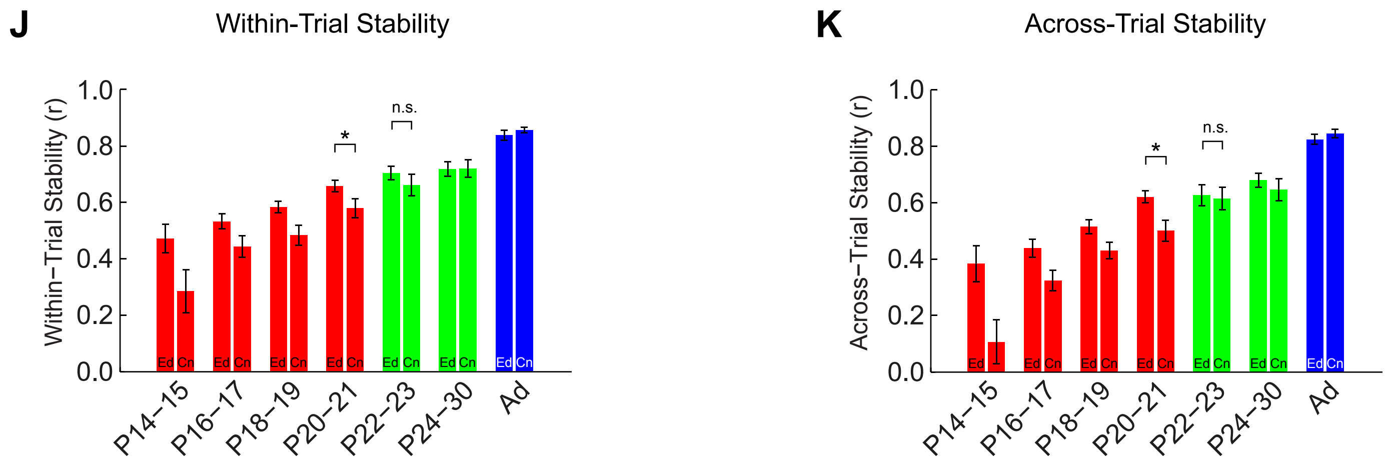

Edge vs centre stability disparity is constant between P16 and weaning age

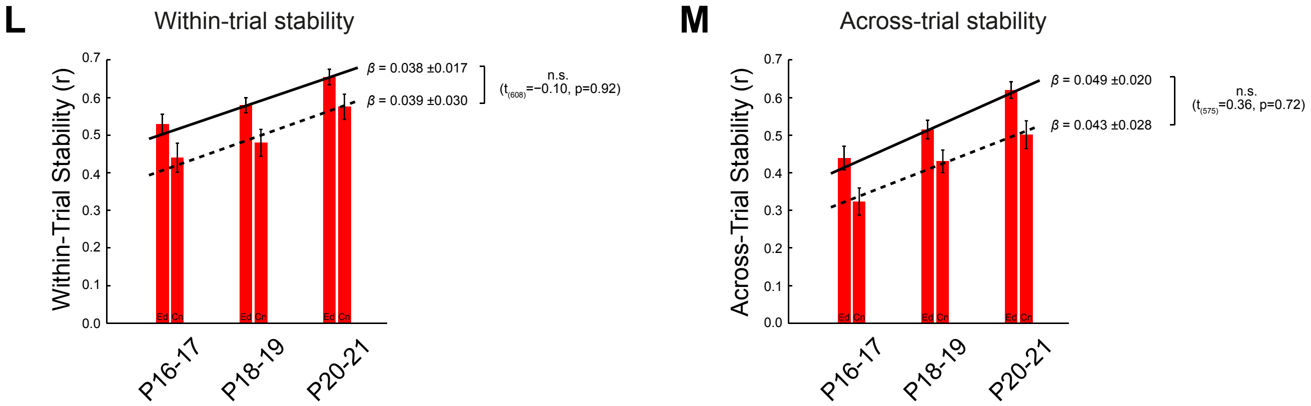

Edge vs centre stability disparity does reduce, abruptly, at weaning age

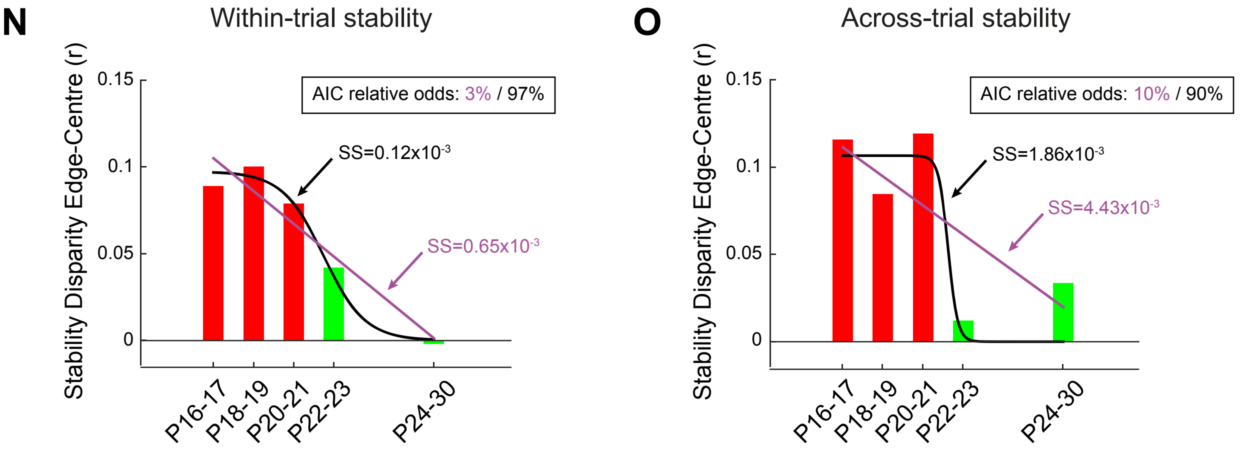

Abrupt emergence of grid cells at weaning age

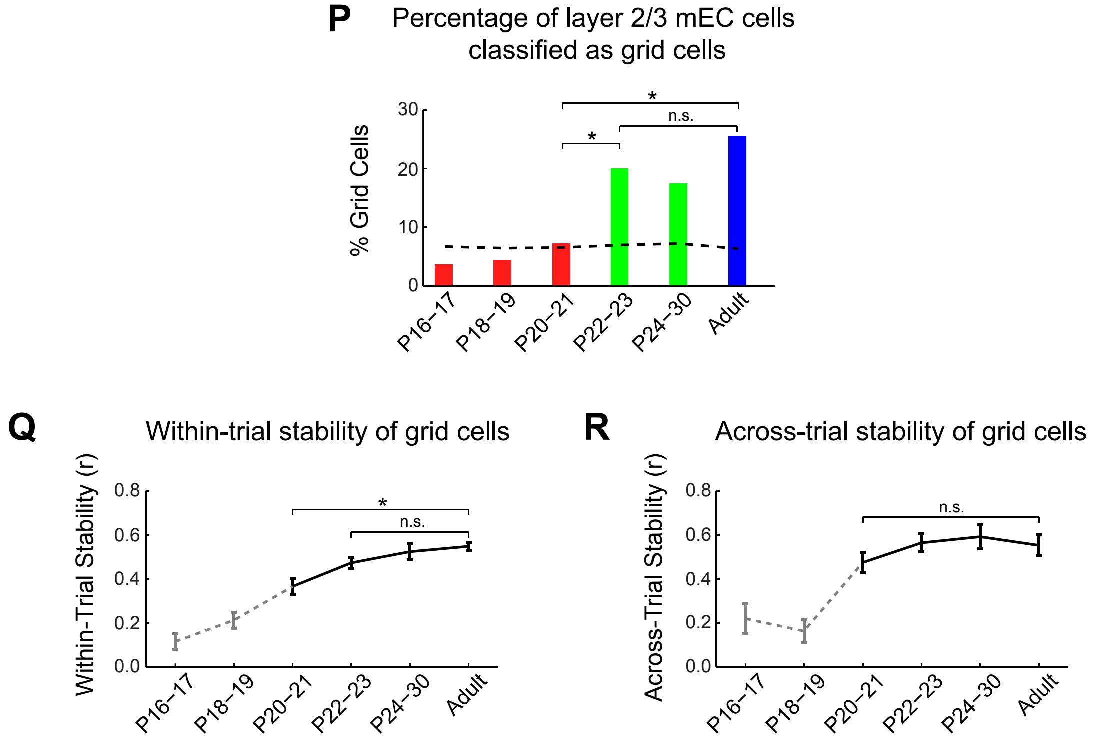

Fig S1: The shift between 'Edge' vs. 'Centre' coding in place cell maps is present in individual animals (A-E) and happens abruptly around weaning (F-O), the same age that a population of grid cells emerges in the superficial layers of the medial entorhinal cortex (P-R). (A-E): Analyses of data obtained from animals where place cells were recorded at least 1 day before and 1 day after weaning. (A) Distributions of place field peaks in pre- and post-weanling animals (7 rats). Mean (±SEM) proportion of place field peaks in 'Edge' zone per animal, pre- and post-weaning (Wilcoxon-Signed-Ranks test, p=0.25). Lines represent data from individual animals. Red line corresponds to animal for which example firing rate maps are shown in (E). (**B**) Place cell firing rates are higher in the edge of environment for pre-weanling animals only (7 rats). Top panel: firing rates for 'Edge' (Ed) and 'Centre' (Cn) zones (mean ± SEM; RM-ANOVA: zone*age: $F_{1,12}$=5.3, p=0.030; SME Zone$_{(pre-wean)}$, p=0.002). Bottom panel: Ratios of firing rates between 'Edge' and 'Centre' zones before and after weaning, within each rat (bottom panel; Wilcoxon-Signed-Ranks test, p=0.043). Key as in (A). * denotes significance at p<0.05. (**C**) Place fields are less stable within-trial in environment centre in pre-weanling animals only (6 rats, as one rat included in A,B had no field peaks in the centre of the environment (see S1A), hence edge versus centre comparisons are not possible). Top panel: Within-trial stability of place cells with peak firing locations in the 'Edge' (Ed) and 'Centre' (Cn) zones of the environment (mean correlation ±SEM; RM-ANOVA: zone*age: $F_{1,10}$=5.2, p=0.048; SME Zone$_{(pre-wean)}$, p=0.045). Bottom panel: Ratios of within-trial stability between 'Edge' and 'Centre' zones before and after weaning, within each rat (Wilcoxon-Signed-Ranks test, p=0.046). Key as in (A). * denotes significance at p<0.05. (**D**) Place fields are less stable across-trial in environment centre in pre-weanling animals only (6 rats). Top panel: Across-trial stability of place cells with peak firing locations in the 'Edge' (Ed) and 'Centre' (Cn) zones of the environment (mean correlation ±SEM; RM-ANOVA: zone*age: $F_{1,10}$=5.1; p=0.047, SME Zone$_{(pre-wean)}$, p=0.016). Bottom panel: Ratios of across-trial stability between 'Edge' and 'Centre' zones before and after weaning, within each rat (Wilcoxon-Signed-Ranks test, p=0.028). Key as in A. * denotes significance at p<0.05 (**E**) False colour firing rate maps for example cells recorded from one animal between P18-23. Within each age group, maps from one cell with peak in Edge (Ed, top) and one with peak in 'Centre' (Cn, bottom) of environment are shown. Leftmost maps ("Within-trial") show whole recording session (first column), first half of session (2nd column) and second half of session (3rd column). Rightmost maps ("Across-trial") show two adjacent recording trials. Numbers top left of maps are peak firing rates (Hz). R-values for within and across trial correlations are shown next to within and across trial maps, respectively. **(F-G) Place cell firing is more concentrated close to environment boundaries in pre-weanling (P14-P21) than in post-weanling (P22-P30) and adult rats. (F)** Distribution of place cell peaks (%) in recording environment shown, at fine timescale, as false-colour quadrant mean maps (corresponding to fig 1B). (**G**) Proportion of place cell peaks in 'Edge' (Ed – bottom portion of each bar) and 'Centre' (Cn – top portion of each bar) zone of the environment at fine timescale (corresponding to fig 1C). Black dashed line indicates value for an even distribution of peaks across zones. The proportion of peaks in the edge zone is significantly greater than the adult proportion at P20-21, but not at P22-23 (Uncorrected p-values from Z-test versus adult proportion: P14-15: Z=1.31, p=0.18; P16-17: Z=2.03, p=0.043; P18-19: Z=1.59, p=0.11; P20-21: Z=2.92, p=0.004; P22-23: Z=0.4, p=0.69; P24-30: Z=-0.22, p=0.83). **(H)** Distribution of place cell firing rates shown, at fine timescale, as quadrant mean rate maps of overall, unsmoothed, firing rate (in Hz) for all recorded place cells in each age group (corresponding to fig 1D). **(I)** Mean place cell firing rate (±SEM) in 'Edge' (Ed) vs. 'Centre' (Cn) zones of environment at fine timescale (corresponding to fig. 1E). There is a significant difference between overall mean rate in the edge and centre zones at P20-21, but not at P22-23 (p-values from uncorrected paired t-tests, full list; P14-15: t=1.96, p=0.057; P16-17: t=1.95, p=0.053; P18-19: t=2.22, p=0.03; P20-21: t=3.58, p<0.001; P22-23: t=0.91, p=0.36; P24-30: t=0.26, p=0.8; adult: t=1.73, p=0.085).**(J-K) Place cell firing is more stable in the 'edge' zone then the 'centre' zone of the environment in pre-weanling (P14-P21), but not post-weanling (P22-P30) and adult rats. (J)** Within-trial stability (mean correlation ±SEM) shown at fine timescale (corresponding to fig. 2A). There is a significant difference between edge and centre stability at P20-21, but not at P22-23 (p-values from uncorrected independent samples t-tests, full list of values; P14-15: t=2.02, p=0.051; P16-17: t=1.9, p=0.06; P18-19: t=2.63, p=0.009; P20-21: t=2.59, p=0.01; P22-23: t=0.97, p=0.34; P24-30: t=0.25, p=0.8; adult: t=0.22, p=0.83). (**K**) Across-trial stability (mean correlation ±SEM) shown at fine timescale (corresponding to fig. 2D). There is a significant difference between edge and centre stability at P20-21, but not at P22-23 (p-values from uncorrected independent samples t-tests, full list of values; P14-15: t=2.75, p=0.01; P16-17: t=2.51, p=0.01; P18-19: t=2.68, p=0.008; P20-21: t=3.07, p=0.003; P22-23: t=0.4, p=0.69; P24-30: t=0.52, p=0.6; adult: t=0.3, p=0.77). **(L-M) The disparity in stability between place cells in the 'edge' and 'centre' zones is constant from P16, until weaning age.** Linear regression was performed on

stability versus age, separately for all 'edge' and all 'centre' cells recorded between P16 and P21. Solid black lines show the best fit for 'edge' place cells, black dashed lines show the best fit for 'centre' place cells. Red bars show the age bin means and SEMs for P16 – P21 (same data as S1J, K), for reference. The slopes for the 'edge' and 'centre' regressions are extremely similar (slope constants β (±95% CI) and statistical significance for slope difference shown on graphs, see also supplemental methods), demonstrating that the reduction of stability in the centre of the environment, relative to the edge, does not change before P22, when grid cells emerge. The youngest rats (P14-15) were not included, as these animals do show an additional sharp change in stability (see S1J,K). This change is likely due to vision onset (median eye-opening age = P15), which marks a significant developmental discontinuity, both in terms of sensory inputs available to the hippocampus, and for the development of the head-direction cell system (Tan et al., 2015). **(N-O) The disparity in stability between place cells in the 'edge' and 'centre' zones reduces abruptly at weaning age.** Red and green bars show the disparity between the mean stability of 'edge' place cells and the mean stability of 'centre' place cells at age bins P16 – P30 (c.f. figure S1J,K). The black lines show the best fits of a logistic function to these data, the magenta lines show the best fits of a straight line (to equate the number of free parameters between the fits, the logistic function was constrained by our prior hypothesis such that (1) the inflexion point was at weaning age, (2) the difference between 'edge' and 'centre' stability for post-weanling data was zero, see supplemental methods for details). The Sum of Squares of the residuals ('SS') for these fits are shown on the graphs: in both cases, the logistic function has a lower SS than the straight line. We used the Akaike Information Criterion (AIC; see supplemental methods) to assess the relative likelihood of the logistic and straight line fits being correct (see 'AIC relative odds' on graphs), in both cases the logistic function was more likely (relative odds: within-trial, 3% vs 97%, across-trial, 10% vs 90%). The improvement of stability in the centre of the environment relative to the edge between P16 and P30 is therefore much better modelled by an abrupt process centred at weaning than a gradual process occurring throughout this period. **(P-R) Abrupt emergence of grid cells at weaning age. (P)** Percentage of superficial mEC cells classified as grid cells across development. Grid cells were defined following (Wills et al., 2010) but using the spatial binning and smoothing parameters used in the current study. The black dashed line shows the 95% confidence level for the percentage of grid cells expected in spatially randomised data. The percentage of grid cells at P20-21 is significantly different to that of P22-23 and that of adults (2-sample Z-test: P20-21 versus P22-23, $Z=11.8$, $p<0.001$; P20-21 versus Ad, $Z=15.2$, $p<0.001$) but the percentage of grid cells at P22-23 is not significantly different to that of adults ($Z=1.01$, $p=0.31$). **(Q, R)** Mean stability of superficial mEC grid cells (±SEM) across ages. Across-trial stability is not significantly different from adult by P20, within-trial stability by P22 (Tukey HSD, sig. threshold $p=0.05$). Lines are greyed-out between P16-P19 as the percentage of grid cells is less than expected in spatially randomised data: cells classified as grids at these ages are treated as false-positives. Panels (P-R) are a re-analysis of previously published data (Wills et al., 2010).

Figure S2 (related to Figure 1 and Figure 2)

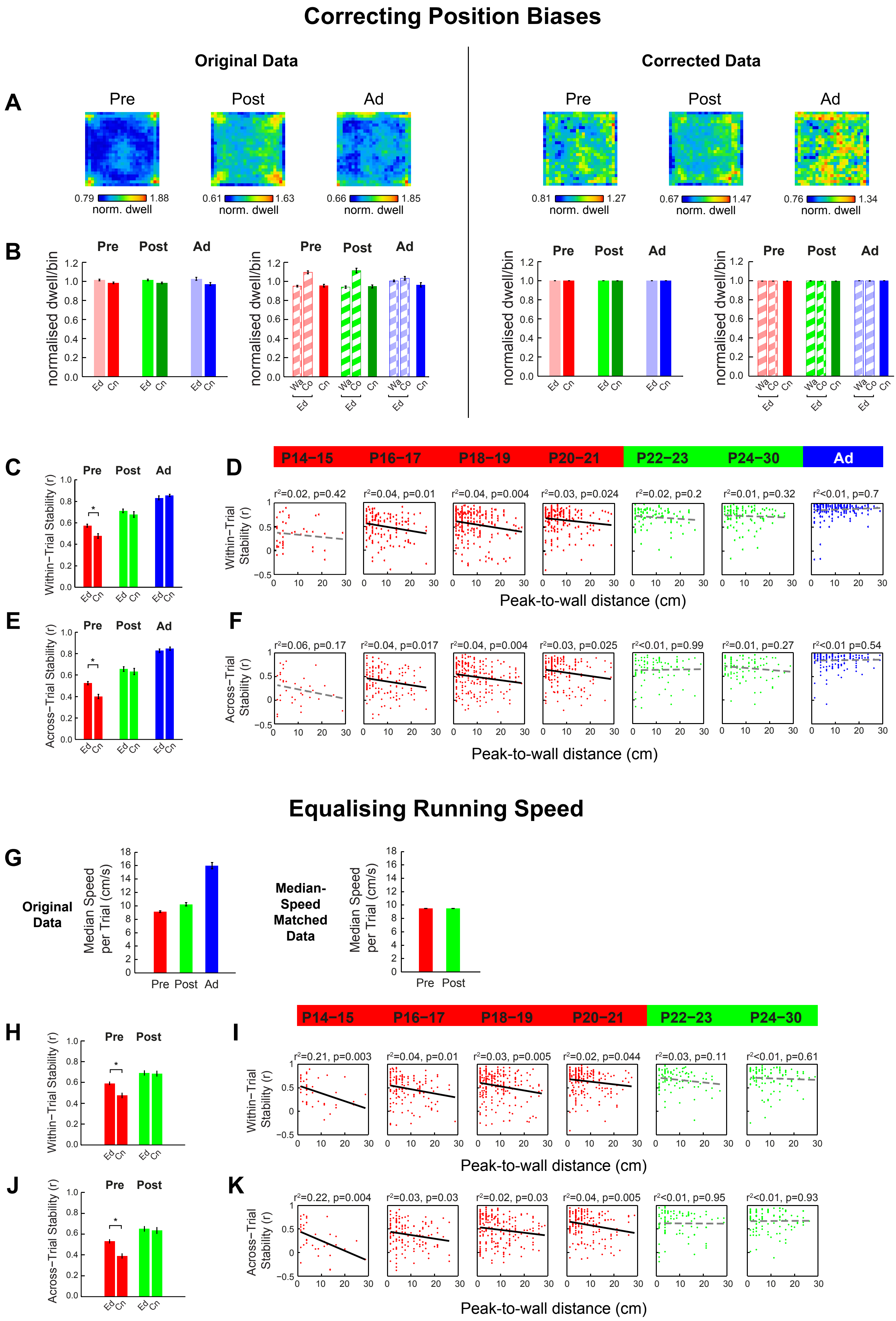

Equalising Firing Rates by Age

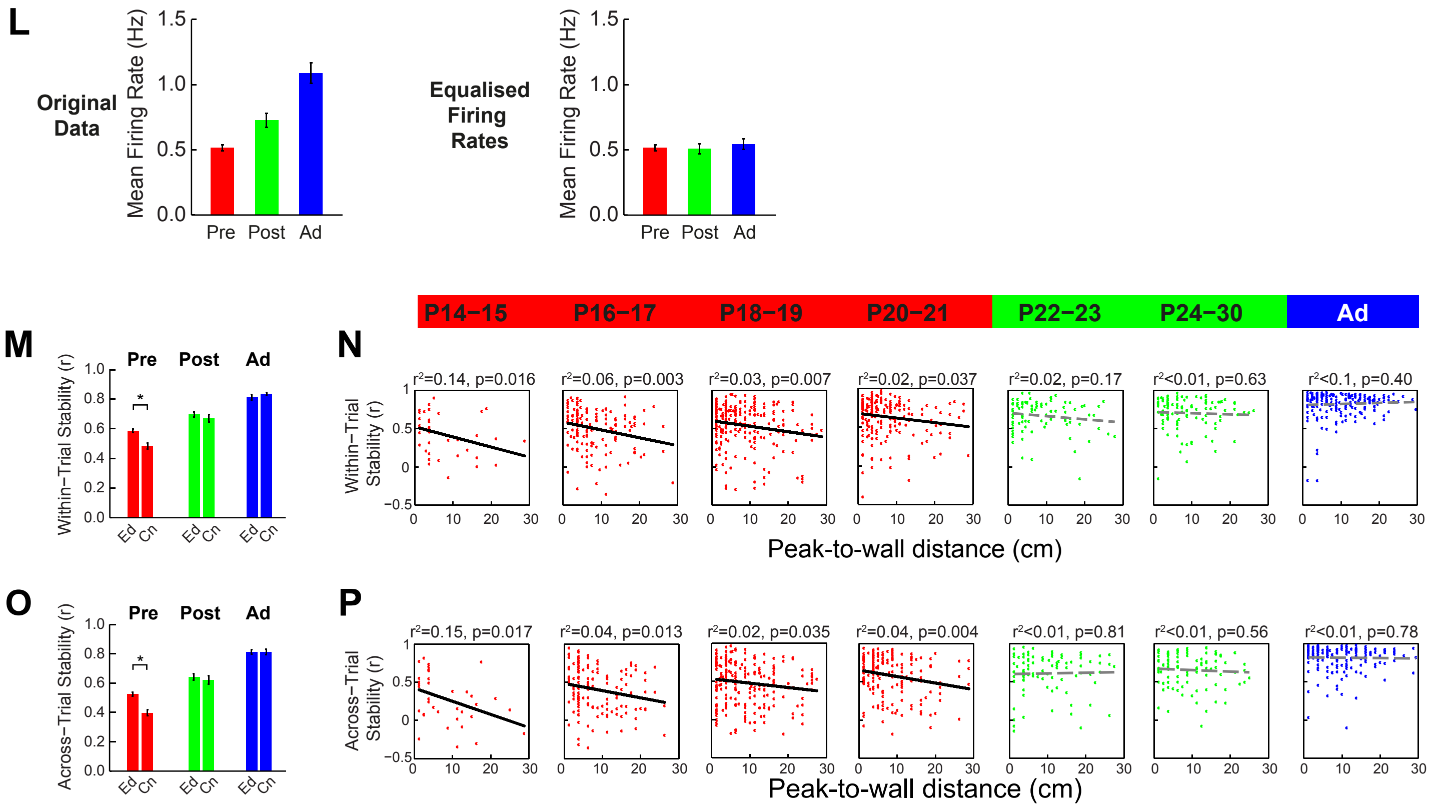

Equalising Firing Rates Across Zones

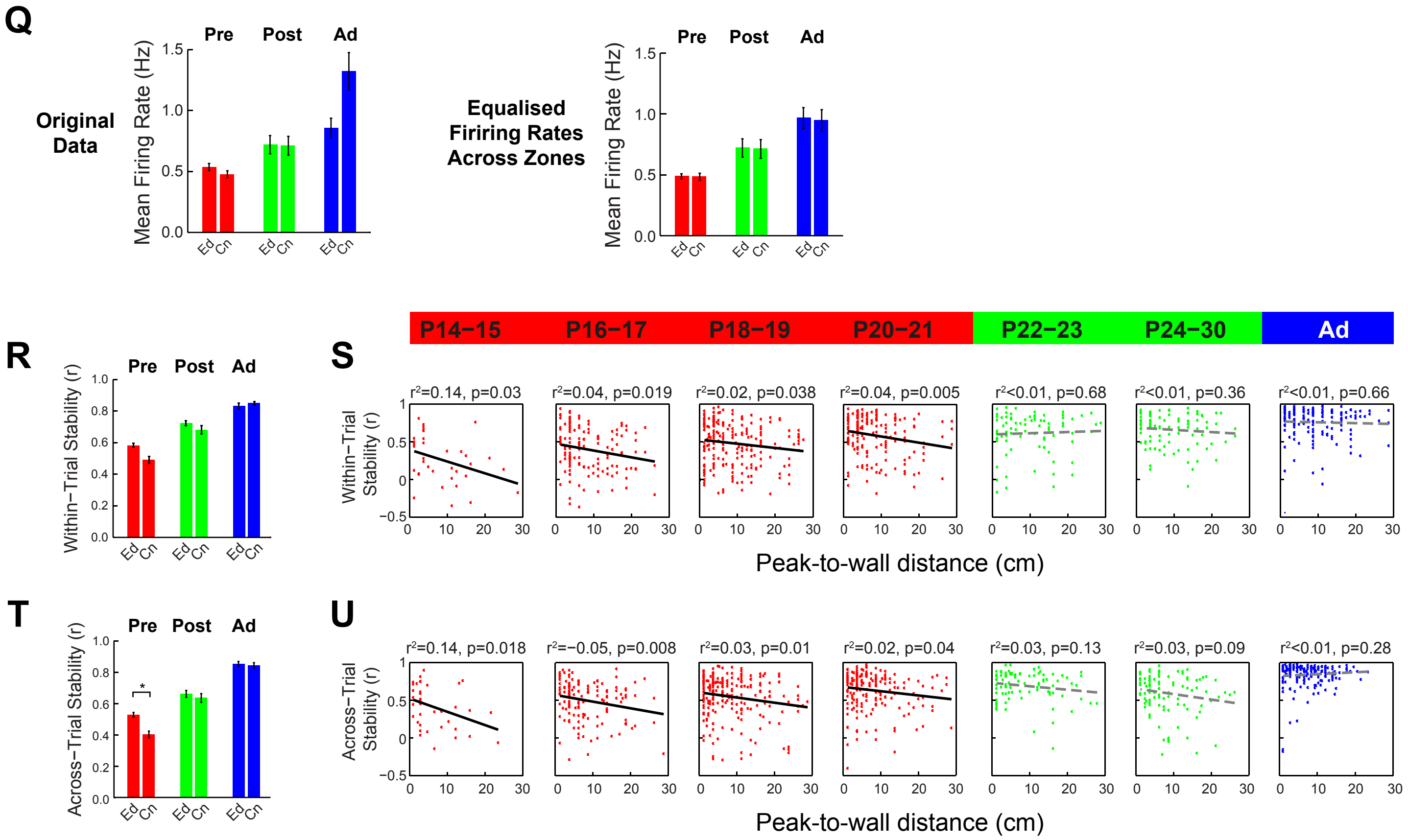

Fig S2: Increased stability of place fields near walls in pre-weanling rats is not related to developmental changes in behaviour or firing rate. (A-F) Place fields are less stable in the centre of the environment in pre-weanling animals, after correcting position sampling biases. (A) False colour maps of mean normalised dwell times for pre-weanling (Pre), post-weanling (Post) and adult (Ad) animals before (left) and after (right) correction of position biases. A normalised dwell of one represents an environment sampled with an equal dwell time in every spatial bin. **(B)** Far left: bar chart depicting mean normalised dwell per bin in the 'Edge' (Ed) and 'Centre' (Cn) zones (±SEM). At all ages, there is a slight bias for greater dwell time in the 'Edge' zone. Second-from-left: mean normalised dwell per bin (±SEM), after sub-dividing the 'Edge' zone into 'Corner' (Co) and 'Wall' (Wa) (see supplemental methods). Rat pups show higher dwell per bin in the corners of the environment, compared to adults (see also (A)). Second-from-right, far right: mean normalised dwell per bin (±SEM) after correction of position biases by down-sampling of data (see supplemental methods for details). **(C)** Within-trial stability of place cells with peak firing locations in the 'Edge' (Ed) and 'Centre' (Cn) zones of the environment, after position biases correction (corresponding to fig. 2A, ANOVA: zone*age: $F_{2,1005}$=3.1, p=0.048; SME $Zone_{(pre-wean)}$, p<0.001). **(D)** Scatter plots of within-trial stability versus distance from peak to nearest wall, after position biases correction (corresponding to fig. 2C). Solid black lines represent linear regression lines of best fit and are significant at p<0.05 level, r^2 and p for regression are indicated above plots. **(E)** Same as (C) but for across-trial stability (corresponding to fig. 2D, F, ANOVA: zone*age: $F_{2,943}$=4.6, p=0.01; SME $Zone_{(pre-wean)}$, p<0.001). **(F)** Same as (D) but for across-trial stability. (**G-K**) **Place fields are less stable in the centre of the environment in pre-weanling animals, after equalising running speed across development. (G)** Left, bar chart depicting median speed per trial (±SEM) of pre-weanling (Pre), post-weanling (Post) and adult (Ad) animals as per original data. Right, bar chart depicting median speed per trial (±SEM) of pre-weanling (Pre) and post-weanling (Post) animals after equalising running speed across these groups (adults not included in analysis, see supplemental methods for details). (**H**): Within-trial stability of place cells with peak firing locations in the 'Edge' (Ed) and 'Centre' (Cn) zones of the environment, after equalising running speed (corresponding to fig. 2A, ANOVA: zone*age: $F_{1,808}$=4.5, p=0.035; SME $Zone_{(pre-wean)}$, p<0.001). (**I**): Scatter plots of within-trial stability versus distance from peak to nearest wall after equalising running speed (corresponding to fig. 2C). Key for plots as in (F). **(J)** Same as (H) but for across-trial stability (corresponding to fig. 2D, F; ANOVA: zone*age: $F_{1,767}$=6, p=0.015; SME $Zone_{(pre-wean)}$, p<0.001). **(K)** Same as (I) but for across-trial stability. (**L-P) Place fields are less stable in the centre of the environment in pre-weanling animals, after equalising firing rates across ages. (L)** Mean firing rates of place cells (±SEM) recorded from pre-weanling (Pre), post-weanling (Post) and adult (Ad) animals before (left) and after (right) firing rate equalisation procedure (see supplemental methods for details). **(M)** Stability within recording trials (mean correlation ±SEM) across age groups in different zones ('Ed' and 'Cn') after firing rates equalisation (corresponding to fig. 2A, ANOVA: zone*age: $F_{2,1005}$=3.4, p=0.033; SME $Zone_{(pre-wean)}$, p<0.001). **(N)** Scatter plots of within-trial stability versus distance from peak to nearest wall after firing rate equalisation (corresponding to fig. 2C). Key for plots as in F. **(O)** Same as (M) but for across-trial stability (corresponding to fig. 2D, F; ANOVA: zone*age: $F_{2,943}$=4.2, p=0.015; SME $Zone_{(pre-wean)}$, p<0.001). **(P)** Same as (N) but for across-trial stability. **(Q-U) Place cell stability, across trials, is lower in the centre of the environment in pre-weanling animals, after equalising firing rates across environment edge and centre zone. (Q)** Mean firing for place cells with peaks in the 'Edge' (Ed) and 'Centre' (Cn) zones of the environment (±SEM) before (left) and after (right) equalisation of firing rates across zones (see supplemental methods for details). **(R)** Within-trial stability of place cells with peak firing locations in the 'Edge' (Ed) and 'Centre' (Cn) zones of the environment, after equalising firing rates across zones (corresponding to fig. 2A). After equalisation, there is no longer a significant interaction between age and zone (ANOVA: zone*age: $F_{2,971}$=2.1, p=0.12). **(S)** Scatter plots of within-trial stability versus distance from peak to nearest wall after equalising firing rates across zones (corresponding to fig. 2C). Key for plots as in (F). **(T)** Same as (R), but for across-trial stability (corresponding to fig. 2D, F; ANOVA: zone*age: $F_{2,911}$=3.4, p=0.036; SME $Zone_{(pre-wean)}$, p<0.001). **(U)** Same as (S), but for across-trial stability.

* denotes significance at p<0.05

Figure S3 (related to Figure 3)

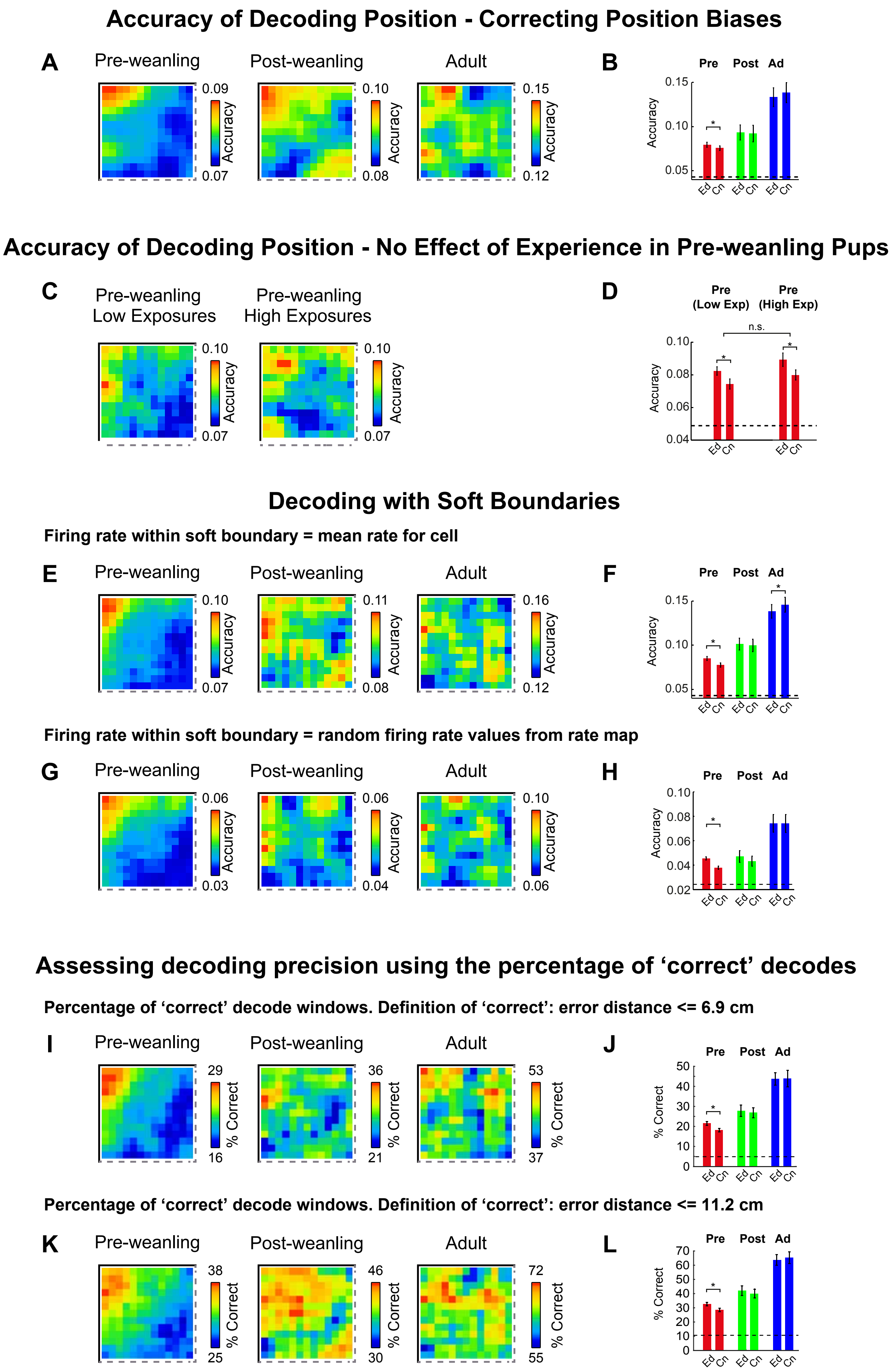

Fig S3: Supplemental Bayesian position decoding analyses. (A, B) Correcting for position biases across development does not affect the differences in decoding accuracy across age groups. Data contributing to the Bayesian decoding analysis was down-sampled such as to equalise the mean dwell time per bin between the 'Edge' and 'Centre' zones (see figure S2A and B; supplemental methods for details of dwell bias and data down-sampling). (**A**) False colour quadrant mean maps of reconstruction accuracy for each age group after correcting position biases (corresponding to fig. 3C). (**B**) Mean accuracy in 'Edge' (Ed) and 'Centre' (Cn) zones of the environment (±SEM). Black dashed line indicates mean expected accuracy from decoding spatially shuffled data. Reconstruction accuracy is higher near to boundaries in pre-weanling, but not in post-weanling or adult rats (corresponding to fig. 3D; ANOVA, Age*Zone, $F_{2,73}$=4.2, p=0.019, SME $Zone_{(pre\text{-}wean)}$ p<0.001, SME $Zone_{(post\text{-}wean)}$, p=0.62, SME $Zone_{(adult)}$, p=0.07). **(C, D) Increased decoding accuracy close to walls in pre-weanling pups is not affected by the number of previous exposures to the recording environment.** The data shown are filtered by whether the rat had either a low or a high number of previous exposures to the environment when the ensemble was recorded (low: 2 – 4 previous exposures, 15 ensembles, median previous exposures = 3; high: 14-26 previous exposures, 14 ensembles, median previous exposures = 18; 2 and 26 represent the absolute minimum and maximum number of previous exposures for the entire pre-weanling dataset). (**C**) False colour quadrant mean maps of reconstruction accuracy for pre-weanling pups, for low or high numbers of previous exposures to the environment. (**D**) Mean accuracy (±SEM) in 'Edge' (Ed) and 'Centre' (Cn) zones of the environment. Black dashed line indicates mean expected accuracy from decoding spatially shuffled data. Reconstruction accuracy is higher near to boundaries for both the low and high exposures groups, but there is no significant effect of the number of exposures (ANOVA, Exp*Zone: Zone, $F_{1,27}$=22, p<0.001; Exp, $F_{1,27}$=2.2, p=0.15; Exp*Zone, $F_{1,27}$=0.14, p=0.72. SME $Zone_{(Low\ Exp)}$, p=0.002, SME $Zone_{(High\ Exp)}$, p=0.004). (**E**-**H**) **Decoding with 'soft boundaries' does not affect accuracy differences between zones across age groups (see supplemental methods for details).** The position decoding analysis was run with 'soft boundaries', whereby the actual environment was embedded into a larger space, into which position could potentially be decoded (the 'soft boundary'). (E, F) and (G, H) differ with respect to the value of the firing rate ascribed to complex spike cells in the soft boundary region. (**E, F**) For each complex spike cell, firing rates ($f_i(x)$) within the soft boundary region were set to the overall mean firing rate for the cell. (**E**) False-colour quadrant mean maps of reconstruction accuracy for each age group (corresponding to fig. 3C). (**F**) Mean accuracy in 'Edge' ('Ed') and 'Centre' ('Cn') zones of the environment (±SEM). Reconstruction accuracy is higher near boundaries in pre-weanling, but not in post-weanling or adult rats (corresponding to fig. 3D; ANOVA Age*Zone, $F_{2,73}$=8.6, p<0.001, SME $Zone_{(pre\text{-}wean)}$ p<0.001, SME $Zone_{(post\text{-}wean)}$, p=0.63, SME $Zone_{(adult)}$, p=0.03). (**G**, **H**) Same as (E, F) but with firing rates for each complex spike cell ($f_i(x)$) in the soft boundary region set to a selection of rate values randomly chosen from the corresponding firing rate map of the actual environment. Note that overall accuracy is reduced, however, the specific increase in accuracy in the 'Edge' zone in pre-weanling rats is preserved (ANOVA, Age*Zone, $F_{2,73}$=3.5, p=0.035, SME $Zone_{(pre\text{-}wean)}$ p<0.001, SME $Zone_{(post\text{-}wean)}$, p=0.16, SME $Zone_{(adult)}$, p=0.99). **(I-L) Assessing decoder precision using the percentage of 'correct' decode windows yields comparable results to accuracy.** The estimate of position in each decode window was classified as 'correct' if the error distance fell below a certain threshold. **(I, J)** Threshold for a correct decode defined as the median adult error distance (6.9cm). **(I)** False-colour quadrant mean maps of the percentage of correct decodes for each age group (corresponding to fig. 3C). **(J)** Mean percentage of correct decodes in 'Edge' ('Ed') and 'Centre' ('Cn') zones of the environment (±SEM). As observed for decoding accuracy, the percentage of correct decode windows is greater in the 'Edge' zone than in the 'Centre' zone, in pre-weanling rats only (corresponding to fig. 3D; ANOVA, Age*Zone, $F_{2,73}$=3.2, p=0.046, SME $Zone_{(pre\text{-}wean)}$ p<0.001, SME $Zone_{(post\text{-}wean)}$, p=0.50, SME $Zone_{(adult)}$, p=0.91). **(K, L)** Same as (I, J) but using the mean adult error distance (11.2cm) as threshold for correct decodes. The percentage of correct decode windows is greater in the 'Edge' zone than in the 'Centre' zone, in pre-weanling rats only (corresponding to fig. 3D; ANOVA Age*Zone, $F_{2,73}$=4.7, p=0.012, SME $Zone_{(pre\text{-}wean)}$ p<0.001, SME $Zone_{(post\text{-}wean)}$, p=0.23, SME $Zone_{(adult)}$, p=0.39).

Supplemental Methods

Analysis of place field position and stability in individual animals. A subset of rats (n=7) contained at least 1 day of recording data both before and after weaning (P21). Data from these animals were analysed in order to assess whether the developmental changes seen in the entire place cell sample were also visible in individual animals. To assess changes in the position of place field peaks, the proportion of peaks in the edge zone was compared pre- and post-weaning, within animal (figure S1A; Wilcoxon signed test). To assess the distribution of place cell firing, the overall mean firing rates for each animal, in the edge and centre, and pre- and post-weaning, were analysed using a repeated measures ANOVA (figure S1B top panel). To further assess trends within animal, the pre- and post-weaning ratios of firing rates in the edge versus the centre were compared (figure S1B bottom panel). To assess trends in stability of place cell firing, a repeated-measures ANOVA was run on the mean stability within each animal (within and across trials) across the two zones of the environment. Additionally we calculated the ratios of correlation coefficients (Z-transformed) between 'Edge' and 'Centre' before and after weaning for each animal. Average ratios before and after weaning were compared with a Wilcoxon-Signed-Ranks test.

Linear regression of age versus stability for edge and centre cells. To assess and compare developmental trends in stability for cells in the 'edge' and 'centre' zones, separate linear regressions were performed for each zone, were the independent variable was defined as the age of the rat (in post-natal days) and the dependent variable was the stability (either across- or within-trial) of the cell. The difference between the 'edge' and 'centre' regression slopes was tested by a t-test, were t was defined as:

$$t = \frac{\beta_1 - \beta_2}{SE_{b1-b2}}$$

where β_1 and β_2 are the slopes of the 'edge' and 'centre' regression coefficients, SE_{b1-b2} is the standard error of the difference between the coefficients, and the degrees of freedom are equal to $n_1 + n_2 - 4$, where n_1 and n_2 are the numbers of cells in the edge and centre regressions, respectively. See (Zar, 2010) for further details. The same method was used to compare the slopes of the stability versus distance-to-wall regressions, see main manuscript figure 2.

Line-fitting the changes in edge-centre stability disparity over age. To further investigate the developmental trends in the disparity between edge and centre stability, we tested whether changes in disparity were better described by functions modelling either a gradual, or an abrupt change. To do this, we found the best fit to the data (that which minimised the sum of the squared Y-residuals) for two functions:

(1) A straight line function, describing a gradual change over age:

$$Y = A + BX$$

where B represents the slope of the line, and A represents the intercept between the line and the line defined by x=0 (i.e. equivalent to linear regression).

(2) A logistic function, which describes an abrupt or sigmoid change over age:

$$Y = A + \frac{(B - A)}{1 + e^{-k(X_0 - X)}}$$

where B represents the upper asymptote of the curve (difference pre-weaning), A represents the lower asymptote of the curve (difference post-weaning), X_0 represents the inflexion point of the curve, and k represents the steepness of the curve. To equate the number of free parameters between the functions, two of the four variables defining the logistic function were constrained by our prior hypothesis to fixed values: the lower asymptote of the curve, A, was set to zero (equivalent to no difference between edge and centre stability after weaning); and the inflexion point of the curve, X_0, was set to weaning age, reflecting our hypothesis that any changes in place cell stability are related to grid cell emergence. The best fit to the data for the parameters k and B (the steepness of the change, and the stability disparity for pre-weanling animals, respectively) were then defined iteratively (using the Matlab NLINFIT function).

For both functions, Y is defined as the difference between the mean 'edge' stability and the mean 'centre' stability in that age bin (for both within- and across-trial stability), and X is defined as age (mid-point of age bin). The fit of these two functions was then compared by examining the sum of squares of the Y-residuals (SS): for both within- and across-trial stability, the SS was smaller for the logistic fit than the linear fit, indicating that the data is better described by an abrupt rather than a linear developmental process. To further quantify the extent to which the logistic function was a better fit to the data, we used the Akaike Information Criterion (AIC), an information theory-based measure which can be used to estimate the relative goodness-of-fit of two models, see (Burnham and Anderson, 2002) for further details. The AIC (corrected for small samples) for each line fitting was defined as:

$$AIC = N.\ln\left(\frac{SS}{N}\right) + 2K + \frac{2K(K+1)}{N-K-1}$$

Where N is the number of data points, SS is the sum of the squared residuals and K is the number of parameters in the model, plus one (as the error in the residuals counts as a model parameter). A smaller AIC corresponds to a better fit. Then, the relative probability, P, that the model with the larger SS is the correct model for the data, relative to the model with the smaller SS, is defined as:

$$P = \frac{e^{-0.5\Delta}}{1 + e^{-0.5\Delta}}$$

Where Δ is the difference between the AICs for the model with the larger SS, and the model with the smaller SS, respectively.

Equalising dwell time between the edge and centre of the environment. Data was sub-sampled so as to equalise the mean dwell times per spatial bin in the edge (distance to nearest wall <= 10cm) and centre (distance to nearest wall > 10cm) zones of the environment. As inspection of the mean dwell time maps showed that the prevalent position bias was for rats to spend more time in the corners of the environment (see figure S2A), for the purposes of sub-sampling, the 'edge' zone was further sub-divided into 'corner' (distances to two nearest walls were both <= 10cm) and 'wall' (distance to the nearest wall <= 10cm, but the distance to the second nearest wall > 10cm) zones. Mean dwell time per spatial bin was equalised between 'centre', 'corner' and 'wall', which also resulted in an equalisation of dwell time between the 'centre' and 'edge' zones used for all main analyses.

For each recording trial that contributed to the analysis, the dwell time per spatial bin in each zone was equalised using the following method: the mean dwell time per spatial bin was calculated for each zone, and the lowest value of these was set as the target dwell per bin for the remaining two zones. In each of the two zones to be sub-sampled, the spatial bins were sorted on the basis of dwell time, and, starting with the bin with the greatest dwell time, a random selection of position samples were removed, so as to match the total dwell time in that spatial bin to the target dwell per bin for the trial. This process was repeated until the *overall* dwell per bin in the zone matched the target dwell per bin for the trial. Spikes occurring within the duration of the discarded position samples were also removed from the data.

For both position sub-sampling analyses presented (Figures S2C-F and S3A,B), the randomised sub-sampling was repeated 100 times, and scores of place cell stability, place cell peak-to-wall distance (S2C-F) and complex-spike ensemble decoding accuracy (S3A,B) were calculated for each repeat. For figure S2C and S2E, the final ANOVA and regressions were performed using the median stability and peak-to-wall distance for each place cell, across the 100 repeated sub-samples. For figure S3B, the final ANOVA was performed on the median accuracy for each ensemble and for each zone ('Edge' versus 'Centre') across the 100 repeated sub-samples. In all cases, the population of cells contributing to the sub-sampled analysis were the same as those for the main analyses. For figure S3A-B, position sub-sampling was applied to the training data for the Bayesian decoder, such that the estimates of probability of being in a given spatial bin ($\mathrm{P}(x)$) and the mean firing rate in each spatial bin ($f_i(x)$) was calculated using data in which dwell time per bin was equalised between the edge, corner and centre zones.

Equalising running speeds between pre- and post-weanling rats. To control for the effect of running speed variation across ages, developmental trends in place cell stability were also analysed after data was filtered so as to match median speed across all pre- and post-weanling animals. The mean of the trial median speeds for all pup data, recorded between P14 and P30, after immobility filtering (speed < 2.5cm/s), was 9.51cm/s. For each individual trial, position and spike data were filtered such that a) all immobility was excluded, b) the median of the remaining speeds was equal

to 9.51cm/s. Thus, for each trial, speed limits s1 > 2.5 cm/s and s2 <∞ were chosen such that the trial median was equal to 9.51cm/s. Spike and position data outside this range were discarded, rate maps were constructed, and analyses of place cell stability were then applied. Adult data were excluded from this control analysis, due to an excessive discrepancy between pup and adult running speeds (see figure S2G).

Equalising place cell firing rates between age groups. To match mean place cell firing rates between pre-weanling, post-weanling and adult groups, the following sub-sampling of spike data was performed. For post-weanling animals 30%, and for adult animals 50% of all recorded spikes from each place cell were removed at random from each cell and the stability of place cells across- and within-trials re-analysed with the sub-sampled datasets. Data recorded from pre-weanling animals was not sub-sampled. This procedure had the effect of matching the overall mean firing rates of the post-weanling, and adult, place cells to the mean firing rate of pre-weanling place cells. The random sub-sampling was repeated 100 times, and scores of place cell stability and place cell peak-to-wall distance were calculated for each repeat. The final ANOVA and regressions were performed using the median stability and peak-to-wall distance for each place cell, across the 100 repeated sub-samples.

Equalising firing rates between the edge and centre of the environment. In order to equalise the firing rates of place cells in the edge and centre zones of the environment, the population of place cells was sub-sampled such that the overall mean firing rate of place cells with peaks located in the edge and centre zones of the environment were matched, within each age group (pre-weanling, post-weanling, adult). Overall mean firing rates for zones were matched by the following method: within each age group, the place cells in each zone were sorted by their firing rate, and the highest rate place cells in the higher mean rate zone, and the lowest rate place cells in the lower rate zone, were removed from the dataset until the mean firing rate for the two zones was matched to within 0.01Hz. Place cells were removed from the two zones in proportion to the sample N in each zone (i.e. if there were twice the number of place cells in the edge zone, two cells would be removed from the edge group for every one from the centre group). After data sub-sampling the analyses performed for figure 2 of the main manuscript were run on the population of remaining cells.

Bayesian decoding with soft boundaries. To investigate whether the geometrical constraints on accuracy near the edges, as opposed to at the centre of the environment affected position decoding accuracy, the decoding was run with ‘soft boundaries’, whereby the actual environment was embedded into a larger space, into which position could potentially be decoded. Before estimation of $P(x|\boldsymbol{n})$, the recorded positions of the rat, x, were shifted by 30cm in both the x and y dimensions, while the maps of both position probability, $\mathrm{P}(x)$, and mean firing rate, $f_i(x)$, were padded with a 12 bin (equivalent to 30cm) wide border on all sides, thus creating a ‘soft boundary’ around the actual visited environment. As $P(\boldsymbol{n}|x)$ is defined for the extent of the firing rate map $f_i(x)$, and $\mathrm{P}(x)$ within

the soft boundary was non-zero (always set to the overall mean dwell probability for the trial), the animal's position could, potentially, be decoded to within the soft boundary. After deriving the most likely estimate of $P(x|\boldsymbol{n})$, the difference between the actual and predicted positions was calculated, and accuracy (1/error+1) derived, as for the main analysis, regardless of whether the decoded position was in the actual environment or in the soft boundary. The quadrant mean maps of accuracy (figure S3E, G) depict only the actual environment, and not the soft boundary, as these maps display the mean accuracy for each *visited* spatial bin, and the soft boundary was not, by definition, visited by the rat. Two different versions of the analysis were run, with different definitions of the mean firing rate $f_i(x)$ in the soft boundary. For figure S3E-F, the firing rate for each cell *i,* for the whole soft boundary region, was defined as the grand mean firing rate for the cell *i* in the actual environment. For figure S3G-H, for each cell *i*, the spatial bins of the soft boundary were set to a random selection of values taken from the firing rate map of cell *i* in the actual environment.

Supplemental References

Burnham, K., and Anderson, D. (2002). Model Selection and Multimodel Inference A Practical Information-Theoretic Approach (New York: Springer-Verlag).

Tan, H.M., Bassett, J.P., O'Keefe, J., Cacucci, F., and Wills, T.J. (2015). The Development of the Head Direction System before Eye Opening in the Rat. Curr. Biol. *25*, 479–483.

Wills, T.J., Cacucci, F., Burgess, N., and O'Keefe, J. (2010). Development of the hippocampal cognitive map in preweanling rats. Science (80-.). *328*, 1573–1576.

Zar, J.H. (2010). Biostatistical Analysis (Prentice Hall).
