# Supplementary material for: A Developmental Switch in Place Cell Accuracy Coincides with Grid Cell Maturation
Source: Neuron. 2015 Jun 3;86(5):1167–73. doi: 10.1016/j.neuron.2015.05.011 (PMC4460188; doi:10.1016/j.neuron.2015.05.011)
Supplement: Document S2. Article plus Supplemental Information [file mmc2.pdf]

Neuron

Report

A Developmental Switch in Place Cell Accuracy Coincides with Grid Cell Maturation

Highlights

- During early development, place cell maps are maximally stable near boundaries
- At weaning age, place cell maps switch to become equally accurate throughout space
- This developmental switch coincides with the emergence of the grid cell network
- Boundary cells may support place maps at edges, and grid cells in the environment center

Authors

Laurenz Muessig, Jonas Hauser, Thomas Joseph Wills, Francesca Cacucci

Correspondence

t.wills@ucl.ac.uk (T.J.W.),
f.cacucci@ucl.ac.uk (F.C.)

In Brief

Muessig et al. show that during early post-natal development, hippocampal place cell maps are maximally accurate near environmental boundaries. Coinciding with grid cell emergence, place maps become accurate throughout space, suggesting that grid cells may stabilize place maps far from boundaries.

CrossMark
Muessig et al., 2015, Neuron *86*, 1167–1173
June 3, 2015 ©2015 The Authors
http://dx.doi.org/10.1016/j.neuron.2015.05.011

CellPress

A Developmental Switch in Place Cell Accuracy Coincides with Grid Cell Maturation

Laurenz Muessig,[1] Jonas Hauser,[1] Thomas Joseph Wills,[2,3,*] and Francesca Cacucci[1,3,*]
[1]Department of Neuroscience, Physiology, and Pharmacology
[2]Department of Cell and Developmental Biology
University College London, Gower Street, London WC1E 6BT, UK
[3]Co-senior author
*Correspondence: t.wills@ucl.ac.uk (T.J.W.), f.cacucci@ucl.ac.uk (F.C.)
http://dx.doi.org/10.1016/j.neuron.2015.05.011
This is an open access article under the CC BY license (http://creativecommons.org/licenses/by/4.0/).

SUMMARY

Place cell firing relies on information about self-motion and the external environment, which may be conveyed by grid and border cells, respectively. Here, we investigate the possible contributions of these cell types to place cell firing, taking advantage of a developmental time window during which stable border cell, but not grid cell, inputs are available. We find that before weaning, the place cell representation of space is denser, more stable, and more accurate close to environmental boundaries. Boundary-responsive neurons such as border cells may, therefore, contribute to stable and accurate place fields in pre-weanling rats. By contrast, place cells become equally stable and accurate throughout the environment after weaning and in adulthood. This developmental switch in place cell accuracy coincides with the emergence of the grid cell network in the entorhinal cortex, raising the possibility that grid cells contribute to stable place fields when an organism is far from environmental boundaries.

INTRODUCTION

Place cells are pyramidal cells in the CA1 and CA3 fields of the hippocampus that fire only when an animal visits selective regions of the environment ("place fields"). Collectively, their firing is thought to constitute a "cognitive map" of an environment, allowing an animal to locate itself and navigate to a goal (O'Keefe and Nadel, 1978).

Place cell firing is thought to integrate inputs from several other types of spatially tuned neurons (Zhang et al., 2013). These include border cells (Solstad et al., 2008), which fire close to the boundaries of an environment, and grid cells (Hafting et al., 2005), which fire in a regular, hexagonally symmetric series of locations across the whole environment; both are found in the medial entorhinal cortex (mEC). Grid cells are thought to encode an intrinsic metric for space based on self-motion information (Burak and Fiete, 2009; Burgess et al., 2007; Fuhs and Touretzky, 2006; Hafting et al., 2005; McNaughton et al., 2006; Zilli and Hasselmo, 2010), whereas boundary-responsive cells such as border cells may, instead, allow external sensory information to stabilize grid and place cell maps near the boundaries of the environment (Burgess et al., 2007; Hartley et al., 2000; Lever et al., 2009; Savelli et al., 2008; Solstad et al., 2008).

Following the discovery of grid cells in the mEC, several theoretical models put forward the hypothesis that place cell firing could be derived solely from grid cell inputs (Fuhs and Touretzky, 2006; Monaco and Abbott, 2011; O'Keefe and Burgess, 2005; Solstad et al., 2006). However, more recent evidence has shown that place fields can exist in the absence of regular grid cell firing both during post-natal development (Langston et al., 2010; Wills et al., 2010) and in adulthood (Koenig et al., 2011). This leaves open the question of the exact contribution of grid cell input to place cell firing.

In this study, we use a developmental model to address this unresolved question. We take advantage of the fact that, during the post-natal development of the hippocampal formation, the first adult-like grid cells emerge at around weaning age (Postnatal day 21 [P21]; Wills et al., 2010), whereas hippocampal CA1 pyramidal cells show spatially tuned and stable firing at least four days earlier, at P16 (Langston et al., 2010; Wills et al., 2010). This developmental timeline provides an opportunity to study the nature of place cell firing before the onset of stable grid cell firing.

A putative stabilizing signal to place cells before grid cells emerge are boundary-responsive cells. In particular, recent work has shown that mEC border cells emerge at P17 and may, therefore, drive stable place cell firing before weaning age (Bjerknes et al., 2014; Wills et al., 2010). We hypothesized that, in pre-weanling animals, when border cells may be the sole stabilizing input to place cells, place fields will be more numerous and more stable close to boundaries. Because of the fact that most boundary-responsive cells are narrowly tuned to locations close to environmental boundaries (Bjerknes et al., 2014; Lever et al., 2009; Solstad et al., 2008; Stewart et al., 2014), place cells should be less stable and less accurate in the center of an open field environment at this age. By contrast, the emergence of stable grid cell firing at weaning age might mark the transition to place cell firing that is stable and accurate throughout the environment.

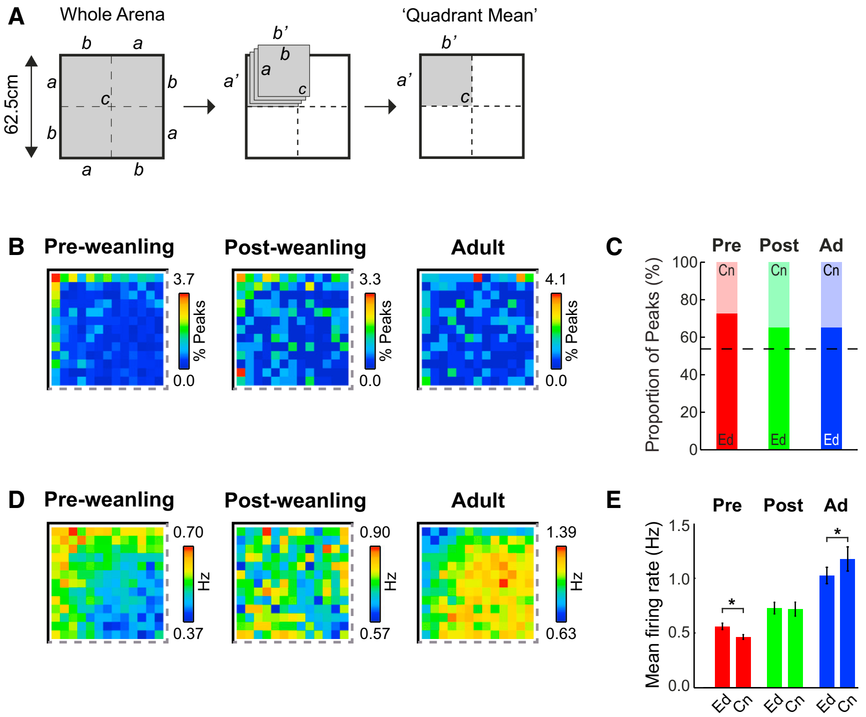

Figure 1. Place Cell Firing Is More Concentrated Close to Environment Boundaries in Pre-weanling (P14–P21) than in Post-weanling (P22–P30) and Adult Rats

(A) Quadrant mean map construction. The full map is divided into quadrants rotated around the center of the environment (*c*) such that all walls *a* are mapped onto *a*' and all walls *b* are mapped onto *b*'.

(B) False-color quadrant mean maps of the distribution of peak firing rate locations (expressed as percent of all peaks).

(C) Proportion of place cell peaks in "edge" ("Ed", bottom part of each bar) versus "center" (Cn, top part of each bar) zones of the environment. The black dashed line indicates the expected proportion for an even distribution of peaks across the environment. Ad, adult.

(D) Quadrant mean rate maps of the overall, unsmoothed firing rate (in Hz) for all recorded place cells in each age group.

(E) Mean place cell firing rate (± SEM) in edge versus center zones of the environment. *p < 0.01 level.

RESULTS

We recorded 813 place cells from the hippocampal CA1 field in pups aged between P14 and P30 and 201 place cells from adult rats under similar conditions (see Experimental Procedures).

An analysis of the positions of place cell firing fields in the recording arena reveals that there is a greater concentration of place fields close to boundaries in pre-weanling pups (P14–P21) compared with post-weanling (P22–P30) or adult rats (Figure 1B; maps are shown in "quadrant mean" format, Figure 1A). To quantify this phenomenon, we calculated the proportion of place cell peaks in two zones of the environment: "edge" and "center" (≤ and >10 cm from the nearest wall, respectively; Figure 1C). All age groups show more place fields in the "edge" zone than expected from an even distribution (e.g., one-sample Z test versus the expected proportion for even distribution; for adults, Z = 3.2, p = 0.001). However, pre-weanling animals show a higher proportion of place fields in the "edge" zone compared with post-weanling or adult rats (Figure 1C; χ^2 test versus equal proportion in all age groups; $\chi^2(2) = 6.53$, p = 0.038; two-sample Z test, pre versus post, Z = 2.04, p = 0.04; two-sample Z test, pre versus adult, Z = 2.04, p = 0.04).

Because many place cells in pre-weanling rats have multiple discrete place fields, we also constructed the mean rate maps of all recorded cells to give a fuller picture of place field location. Place cell firing is concentrated toward the boundaries in pre-weanling animals and toward the environment center in adults, whereas no bias exists in post-weanling rats (Figure 1D). When comparing mean firing rates in the two zones of the environment, we find that firing rates are higher in the "edge" in pre-weanling pups and lower in adults (Figure 1E; ANOVA zone*age $F_{2,1011}$ = 8.8, p < 0.001; post hoc comparison within age group (simple main effects [SMEs]), SME $zone_{(pre\text{-}wean)}$, p = 0.001; SME $zone_{(adult)}$, p = 0.004), with no differences in post-weanling animals (SME $zone_{(post\text{-}wean)}$, p = 0.88). The developmental trends in place cell field position and firing distribution are also visible in individual animals (Figures S1A and S1B; Supplemental Experimental Procedures) and occur abruptly between P20-21 and P22-23 (Figures S1F–S1I), suggesting that a step change in the distribution of the hippocampal representation of space occurs around weaning age. In pre-weanling rats, place cell firing is concentrated near boundaries, consistent with the hypothesis that, at this age, place cells receive spatial input from border cells.

Correspondingly, we found that, only in pre-weanling animals, place fields closer to environmental walls ("edge" zone) are significantly more stable than those located in the middle of the environment ("center" zone; Figures 2A and 2B; within-trial stability, ANOVA age*zone, $F_{2,1005}$ = 3.2, p = 0.042, SME $zone_{(pre\text{-}wean)}$ p < 0.001; see Figure 2B for example place fields). Furthermore, the stability of place fields recorded from pre-weanling rats (but not from post-weanling or adult rats) is inversely correlated to the distance from environmental boundaries (Figure 2C). The regression line slope for all pre-weanling data is also significantly steeper than that for post-weanling data (slope constants: pre, -8.3×10^3; post, -3.3×10^3; t = 1.83, degrees of freedom [df] = 808, p = 0.03 [one-tailed]). In summary, during development, the hippocampal map of space is initially only stable close to environmental boundaries and becomes as stable away from these boundaries from weaning age onward.

We tested whether the inhomogeneity in within-trial stability would also apply to across-trial stability. We found that place fields near walls have greater across-trial stability in

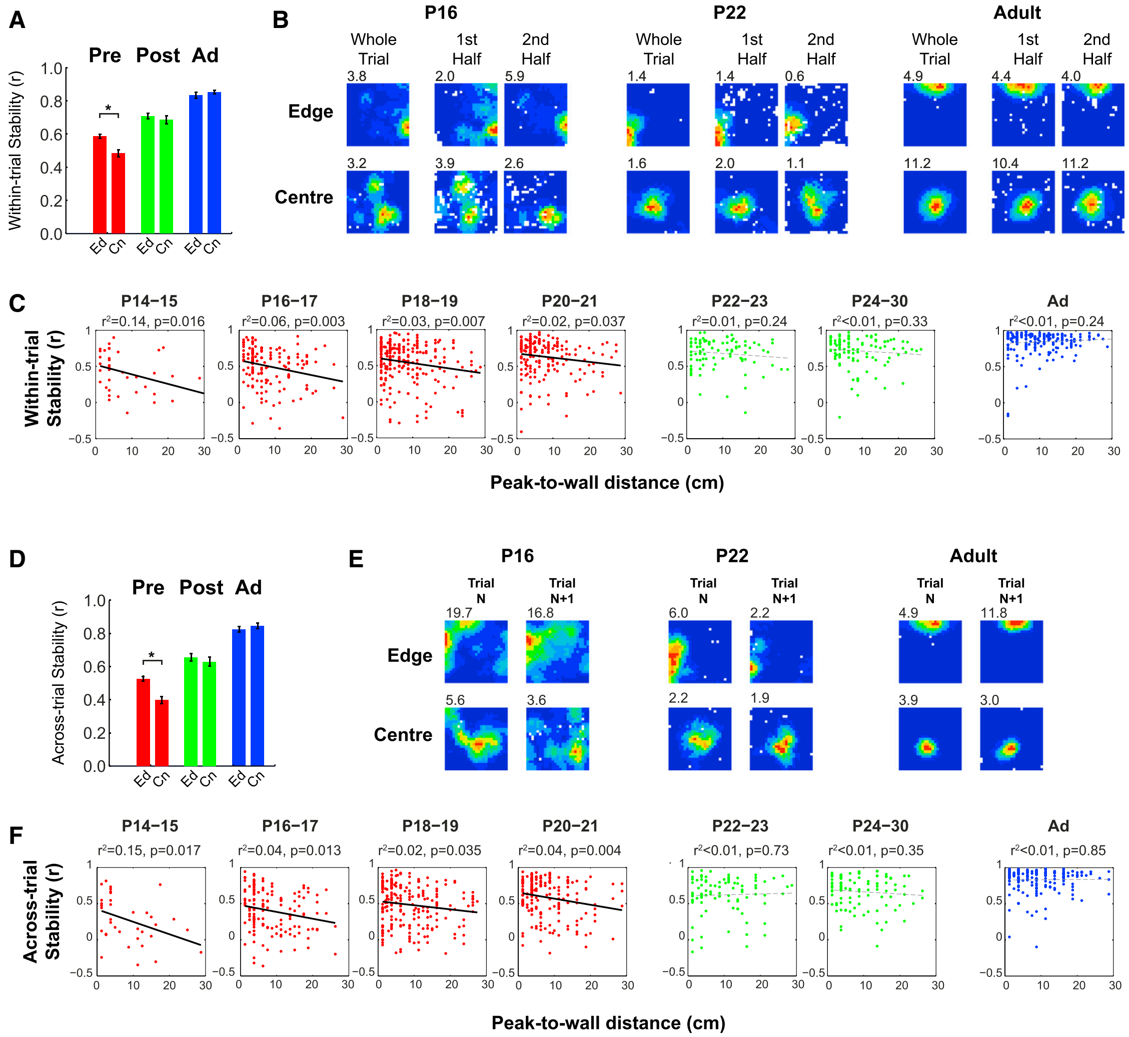

Figure 2. Place Fields Are More Stable Close to Environmental Walls in Pre-weanling Pups

(A) Mean within-trial stability (± SEM) of place cells with peak firing locations in the edge and center zones of the environment.

(B) False-color firing rate maps from representative example place cells showing within-trial stability at P16, P22, and adult. Within each age group, the maps show, from left to right, the whole recording session, the first half of the session, and the second half of the session for place fields with firing peaks located close (top) or far (bottom) from a wall (stability values for examples lie within SD of the mean of the respective population).

(C) Scatterplots of within-trial stability versus distance from the peak to the nearest wall with linear regression lines of best fit. Solid black lines are significant at the $p < 0.05$ level, and r^2 and p for regression are shown above the plots.

(D) Mean across-trial stability (± SEM) of place fields with peak firing locations in the edge and center zones.

(E) Firing rate maps showing example across-trial stability at P16, P22, and adult. Within age groups, the left and right columns show two recording sessions separated by 15 min.

(F) Scatterplots of across-trial stability versus distance to wall with lines of best fit. Solid black lines are significant at the $p < 0.05$ level, and r^2 and p for regression are shown above the plots.

pre-weanling pups but not in post-weanling or adult animals (Figures 2D and 2E; ANOVA age*zone, $F_{2,943} = 5.6$, $p = 0.004$; SME $zone_{(pre\text{-}wean)}$ $p < 0.001$; SME $zone_{(Post\text{-}wean)}$; $p = 0.46$; SME $zone_{(Adult)}$, $p = 0.76$; see Figure 2E for example place fields). There is a significant inverse relationship between place field stability and distance to wall for all 2-day age groups between P14 and P21, but not for P22 and older (Figure 2F), and the regression slope for all pre-weanling data is significantly steeper than that of post-weanling data (slope constants: pre, -8.6×10^3; post, -0.9×10^3; $t = 2.85$, $df = 767$, $p = 0.004$ [one-tailed]). In conclusion, the pattern of place field stability between different visits to the same environment recapitulates that of within-trial stability. Before weaning, stability is lower further from boundaries, and, after weaning, stability is equal throughout the environment.

The switch between place maps that are selectively more stable close to walls (both within- and across-trial) to ones that are equally stable throughout the environment is also

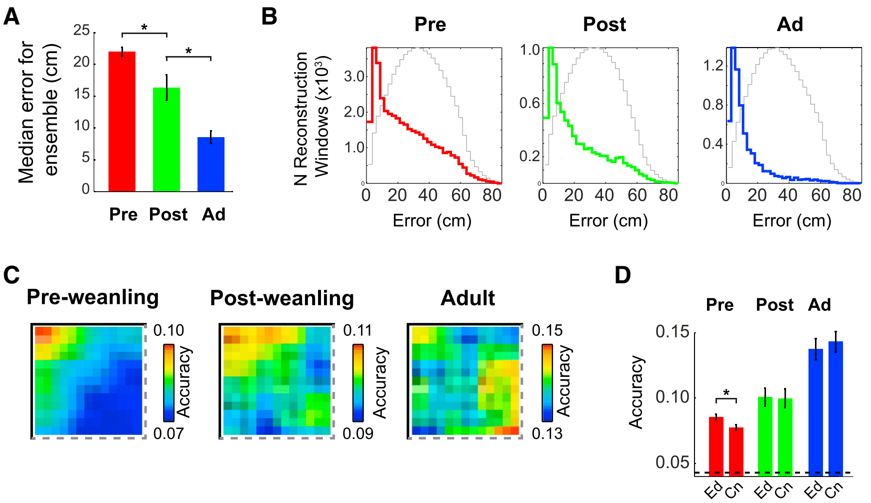

Figure 3. In Pre-weanling Pups, the Accuracy of Position Decoding Is Higher near Environment Boundaries

(A) Median reconstruction error per ensemble for each age group (mean ± SEM). *p < 0.01 level.

(B) Distribution of errors for all 1-s reconstruction time windows in each age group. Colored lines show error distributions for real data, and gray lines show errors from spatially shuffled data (Experimental Procedures).

(C) Quadrant mean false-color heat map of reconstruction accuracy (1 / (error + 1)) for each age group.

(D) Mean accuracy (± SEM) in "edge" and "center" zones of the environment. The black dashed line indicates the mean expected accuracy from decoding spatially shuffled data (Experimental Procedures). *p < 0.01 level.

apparent in individual animals (Figures S1C–S1E) and occurs abruptly between P20-21 and P22-23 (Figures S1J–S1O; Figures 2C and 2F). Furthermore, these developmental changes are independent of other behavioral and physiological changes occurring during the same developmental period (Figure S2; with a single exception, Figure S2R).

To investigate whether the observed differences in place cell firing near and far from boundaries in pre-weanling rats affect the ability of the hippocampus to accurately encode position, we tested whether a Bayesian decoding algorithm (Zhang et al., 1998; Experimental Procedures) could reconstruct the rat's location more accurately close to boundaries in pre-weanling rats from the firing of all recorded CA1 pyramidal cells. First, we established that Bayesian decoding can reconstruct position in developing rats. As expected, the overall reconstruction error is higher in developing than in adult rats (ANOVA age $F_{2,76} = 31$, $p < 0.001$; Tukey honestly significant difference [HSD]; all groups different at $p < 0.01$; Figure 3A), but, for both pre-and post-weanlings, the modal reconstruction error is the same as in adult rats (2.5–5cm), and the distribution of errors is significantly different from that expected from random reconstruction (Kolmogorov-Smirnov [KS] test: pre-wean, k = 0.23, $p < 0.001$; post-wean, k = 0.35, $p < 0.001$; Figure 3B; Experimental Procedures). Examining the spatial biases of decoding error, we found that reconstruction accuracy (1 / (error + 1)) is higher near boundaries in pre-weanling but not in post-weanling or adult rats (Figures 3C and 3D; ANOVA age*zone, $F_{2,73} = 8.5$, $p < 0.001$; SME $zone_{(pre\text{-}wean)}$ $p < 0.001$; SME $zone_{(post\text{-}wean)}$, $p = 0.91$; SME $zone_{(Adult)}$, $p = 0.085$). This result is independent of behavioral biases or the amount of previous experience of the environment (Figures S3A–S3D; Supplemental Experimental Procedures) and is not related to geometrical constraints on accuracy scores at the edge of the environment (Figures S3E–S3L).

In pre-weanling pups, the place cell representation of space affords less accurate self-localization in the center of the environment than near boundaries, whereas, in post-weanling and adult animals, the place cell code is evenly accurate throughout the explored space.

DISCUSSION

We have demonstrated an important developmental step change in the nature of the hippocampal representation of space in rats. Before weaning, the hippocampus encodes space more accurately close to boundaries (where input from border cells would be maximal; Bjerknes et al., 2014), whereas, after weaning, the accuracy of the hippocampal representation of space appears to be even throughout the environment. These findings are independent of physiological and behavioral changes taking place during development, and, therefore, represent a genuine change in hippocampal processing, taking place around weaning age.

This sharp developmental switch coincides with the sudden emergence of a stable grid cell network in the mEC. In animals tested under the same experimental conditions, grid cells first emerge at P20-21, but the proportion of grid cells is extremely low at these ages and significantly less than that observed in the adult. At P22-23, the percentage of mEC cells classified as grid cells suddenly reaches a level that is not significantly different to that observed in the adult (Wills et al., 2010; Figure S1P–S1R). Furthermore, in vitro recordings show that mEC stellate cell network synchronization significantly increases at P22 (Langston et al., 2010). This suggests that the widespread recurrent network thought to be necessary for grid cell activity (Burak and Fiete, 2009; Bush and Burgess, 2014; Fuhs and Touretzky, 2006; McNaughton et al., 2006; Zilli and Hasselmo, 2010) emerges at this age.

We interpret our results to suggest that grid cells may be necessary to provide a stable and accurate representation of position throughout an environment when the organism is far from environmental landmarks or boundaries. When place cells do not receive grid cell input, as in pre-weanling pups, error in their estimate of location increases when animals are further from boundaries. This hypothesized gain of function grid cells would provide to place cells is consistent with their widely proposed role in path integration (Burak and Fiete, 2009; Burgess et al., 2007; Fuhs and Touretzky, 2006; Hafting et al., 2005; Hasselmo et al., 2007; McNaughton et al., 2006). By calculating an

estimate of position on the basis of self-motion cues, the role of grid cells may be to allow an accurate representation of position even when environmental cues are relatively sparse, for example, in darkness or in the center of an open field environment (Bush et al., 2014; Poucet et al., 2014). This interpretation is also consistent with recent evidence from adult rats undergoing medial septum inactivation (which disrupts theta sequences and grid cell firing; Brandon et al., 2011; Koenig et al., 2011; Wang et al., 2015) while exploring a large novel environment. Most CA1 cells did not exhibit spatial firing, but those that did had place fields at the edges of the environment (Wang et al., 2015).

Alternative explanations of our findings could involve intra-hippocampal mechanisms such as changes in synaptic plasticity (Blair et al., 2013). The age at which theta sequences emerge remains unknown, but the distribution of place fields in adults with disrupted theta sequences (Wang et al., 2015) might suggest this as another candidate explanation. Alternatively, it is possible that a single mechanism might underlie both the emergence of grid cells and the stabilization of place maps observed at weaning. For example, at weaning, the proportion of theta-modulated cells in CA1 and entorhinal cortex reaches adult values (Wills et al., 2010). The maturation of theta rhythmicity at weaning might underlie the stabilization of place fields into open space, either directly or indirectly by spurring the emergence of grid cells in the entorhinal cortex.

We have demonstrated that, at ages when border cells are present (Bjerknes et al., 2014) but grid cells have yet to emerge (Wills et al., 2010), place cells are more numerous and more stable close to boundaries. This developmental pattern may provide the first experimental evidence for a further hypothesis: that boundary-responsive cells such as border cells or boundary-vector cells (Hartley et al., 2000; Lever et al., 2009) "anchor" place and grid cell maps by providing a stabilizing input when an animal is close to environmental boundaries (Burgess et al., 2007; Savelli et al., 2008; Solstad et al., 2008). The concentration of place cell fields close to boundaries in pre-weanling pups is also consistent with the theory that place fields may be constructed from inputs from boundary-responsive cells (Hartley et al., 2000). Our results also suggest that boundary-responsive cells may be a foundational spatial signal (Bjerknes et al., 2014; Wills et al., 2010; F. Cacucci et al., 2013, Soc. Neurosci., abstract 485.16), along with head direction responses (Langston et al., 2010; Taube et al., 1990; Wills et al., 2010), during the ontogeny of hippocampal spatial representations.

Our results also offer a functional explanation as to why hippocampus-dependent behavior emerges around weaning age in rats: the first evidence of learning on spatial memory tasks appears at this age (Green and Stanton, 1989; Rauch and Raskin, 1984; Rudy et al., 1987; Schenk, 1985). Given the relationship between place cell firing and spatial behavior in adult rats (Lenck-Santini et al., 2002; O'Keefe and Speakman, 1987), this behavioral transition seems likely to be due to place cell maturation. However, previous studies of hippocampal development (Langston et al., 2010; Scott et al., 2011; Wills et al., 2010) have failed to find any candidate sharp changes in the functional properties of pre- and post-weanling place cells that might underlie the switch to a behaviorally functional navigation system. Our data show such a functional step change in CA1 place cells occurring precisely at weaning age, suggesting that the hippocampus supports spatial learning and memory only after the emergence of a cognitive map equally stable and accurate throughout an environment. This, in turn, may rely on the emergence of a grid cell network in the mEC.

EXPERIMENTAL PROCEDURES

Subjects

43 male Lister Hooded rat pups, aged P12–P22 and weighing 24–64 g on the day of surgery, were used as subjects. Litters were bred in-house and remained with their dams until weaning (P21). Rats were maintained on a 12:12 hr light:dark schedule (lights off at 12:00). At P4, litters were culled to 8 pups/dam to minimize inter-litter variability. After surgery, each pup was separated from the mother for 30 min to 2 hr per day to allow for electrophysiological recordings. 13 male Lister Hooded adult rats, aged 4–6 months at the time of recording, were included in the study to provide a comparison for the pup data. Data from 17 of the subjects in this study (14 rat pups, 368/813 place cells; 3 adult rats, 43/201 place cells) have also contributed toward a previously published study (Wills et al., 2010). There were no differences in procedure between the two groups. The methods set out below apply equally to both groups of rats. All experiments were carried out in accordance with the relevant UK legislation (ASPA 1986).

Surgery and Electrode Implantation

Rats were anesthetized using 1%–2% isoflurane and 0.15 mg/kg body weight buprenorphine. Rats were chronically implanted with microdrives loaded with 4-8 tetrodes (HM-L-coated 90% platinum/10% iridium 17-μm-diameter wire) aimed at the hippocampal CA1 region (2.9 mm posterior and 1.8 mm lateral to bregma). After surgery, rats recovered in a heated chamber (10–30 min) and were then returned to their mothers.

Single-Unit Recording

Rats were allowed 1 day of postoperative recovery, after which electrodes were advanced by 62–250 μm/day until the CA1 pyramidal layer was identified by the presence of complex spike cells and 200-Hz "ripple" fast oscillations. At this point, recording sessions began. Single-unit data were acquired using an Axona DACQ system. Light-emitting diodes (LEDs) were used to track the position and directional heading of the animal. Isolation of single units from multi-unit data was performed manually on the basis of peak-to-trough amplitude using the software package TINT (Axona). Isolated single units were only used for further analysis if they fired ≥ 75 spikes in a trial.

Classification of Single Units as Complex Spike Cells

Single units recorded in the CA1 were classified into complex spike cells (putative pyramidal cells) and putative interneurons using k-means clustering based on the following parameters: spike width (peak to trough); burst-firing at 3–10 ms, as assessed by the first moment of the temporal autocorrelogram, within a 50-ms window; and the mean firing rate of the cell (Csicsvari et al., 1999). If a cell was recorded on multiple trials, the trial with the highest mean rate was used to define these values. Because the physiological properties of CA1 neurons change during development (Wills et al., 2010), adult and pup data were clustered separately.

Behavioral Testing

Single-unit activity was recorded while rats searched for drops of soya-based infant formula milk randomly scattered in a square, light gray wooden box (walls, 62.5 cm long and 50 cm high) placed on a black plastic platform. Trials were 10–15 min long. The fixed apparatus of the laboratory provided distal visual cues. Rats were kept in a separate holding box between recording trials (inter-trial interval, 15 min). Each rat was given between 1–4 recording trials per session. The median number of previous exposures to the recording environment was 11 (minimum = 0; maximum = 44; quartile range, 6–18).

Construction of Firing Rate Maps

The edges of the visited environment were defined as the line of camera pixels (2.5 mm wide) furthest from the center of the environment where the total dwell time was ≥ 1 s. Positional data within the visited edges of the environment were then sorted into 2.5 × 2.5 cm spatial bins. Data were included in further analyses only if total path length >45 m and the rat visited $\geq 94\%$ of the total surface area of the arena (≥ 585 of 625 total spatial bins). All spike and positional data were filtered to remove periods of immobility (speed, <2.5 cm/s for pups and <5 cm/s for adults). The total dwell time and spike count for the whole trial were then calculated for each spatial bin. The binned data were then smoothed using adaptive smoothing (Skaggs et al., 1996). In brief, to calculate the firing rate for a given bin, a circle centered on the bin was gradually expanded in radius *r* until

$$r \geq \frac{\alpha}{d\sqrt{s}},$$

where $\alpha = 200$ and *d* and *s* are the dwell time (in seconds) and the number of spikes lying within the circle, respectively. The firing rate assigned to the bin was then set equal to *s/d*. The exception to this procedure consisted of the overall mean rate maps for all cells (Figures 1D and 1E); here, no smoothing was applied.

Criteria for Classification of Place Cells

Complex spike cells were classified as place cells on the basis of the spatial information of their rate maps, expressing the extent to which a cell's firing can be used to predict the position of the animal. The estimate of the mutual information I(R|X) between the firing rate R and location X is

$$I(R|X) \approx \sum_i p(\vec{x}_i) f(\vec{x}_i) \log_2 \left(\frac{f(\vec{x}_i)}{F} \right),$$

where $p(\vec{x}_i)$ is the probability for the animal being at location $\vec{x}_i$, $f(\vec{x}_i)$ is the firing rate observed at $\vec{x}_i$, and F is the overall firing rate of the cell (Skaggs et al., 1996). I(R|X) was then divided by the mean firing rate of the cell, giving an estimate in bits/spike. Cells were classified as place cells if their spatial information exceeded a threshold defined as the 95th percentile of a population of spatial information scores derived from age-matched, spatially shuffled data (Wills et al., 2010).

Quantification of Place Field Position and Stability

Place field location was defined as the position of the peak rate pixel in the rate map. Field-to-wall distance was defined as the minimum distance from the field peak to the environment edges. Across-trial stability was defined as the correlation (Pearson's r) between spatially corresponding bins from two consecutive trials, excluding bins with a firing rate of 0 Hz in both trials. Trial pairs were used if a complex spike cell was classified as a place cell on at least one of the trials. Within-trial stability was the correlation between the spatially corresponding bins of the rate maps from the temporal first and last halves of the trial. If a place cell was recorded for more than one trial, the stability and peak-to-wall distance for that cell were defined as the mean over all trials recorded. Stability is displayed as r values; however, these were Z-transformed for ANOVAs. For analysis of peak position, to avoid the centralizing tendency of averaging peak positions, only one trial for each cell was used: that on which the cell was first defined as a place cell. The difference between stability versus distance-to-wall regression slopes was compared using a t test (Supplemental Experimental Procedures).

Bayesian Reconstruction of Position

The rat's position was reconstructed following (Zhang et al., 1998). For each 1-s reconstruction time window, the probability of the rat being in a spatial bin *x*, given the numbers of spikes fired an ensemble of N cells, represented by the vector $\boldsymbol{n}$, was defined as:

$$P(x|\boldsymbol{n}) = \frac{P(\boldsymbol{n}|x)\mathrm{P}(x)}{P(\boldsymbol{n})}.$$

P(*x*) is the probability that the rat was at position *x*, defined as the ratio between the dwell time for spatial bin *x*, and the length of the trial. $P(\boldsymbol{n}|x)$, the probability of N cells in the ensemble firing *n* spikes in time window T, given that the rat was at position *x*, was derived from the firing rate map for each cell *i* as follows:

$$P(\boldsymbol{n}|x) = \prod_{i=1}^{N} P(n_i|x) = \prod_{i=1}^{N} \frac{(\tau f_i(x))^{n_i}}{ni!} \exp(-\tau f_i(x)),$$

where $f_i(x)$ is the mean firing rate of cell *i* at position *x*, and τ is the length of the time window in seconds. The probability P($\boldsymbol{n}$) was determined by normalizing the conditional probability $P(x|\boldsymbol{n})$ so that the sum of $P(x|\boldsymbol{n})$ over x was equal to 1. The calculation of $P(x|\boldsymbol{n})$ was made with reference to only spiking in the current time window T; i.e., the prior probability distribution on the basis of the previous reconstruction at T_{t-1} was assumed to be flat.

For every time window T, $P(x|\boldsymbol{n})$ was calculated for every spatial bin, and the bin with the maximum *P* was considered to be the reconstructed position. The reconstruction error for T was then defined as the linear distance between the reconstructed position and the mean actual position of the rat during time window T. To determine whether spatial biases exist in the distribution of successful reconstructions, error values were converted to accuracy, *a*, as follows:

$$a = 1/(\mathrm{e} + \varepsilon),$$

where Ɛ is a constant (set to 1 cm) so that very small errors do not have an undue influence on overall accuracy.

The reconstruction analysis was based on the spiking of all recorded complex spike cells (putative pyramidal projection cells) to provide an estimate of the location information available to downstream brain areas. To allow sufficient data for successful reconstruction, only sessions with ≥ 10 complex spike cells were used. Time windows in which mean speed was below the threshold for immobility were excluded. Reconstruction errors expected from spatially shuffled data were generated by randomly reassigning the identities of the rate maps with respect to the spike trains (10,000 times) before applying the decoding algorithm.

SUPPLEMENTAL INFORMATION

Supplemental Information includes Supplemental Experimental Procedures and three figures and can be found with this article online at http://dx.doi.org/10.1016/j.neuron.2015.05.011.

ACKNOWLEDGMENTS

We wish to thank Caswell Barry, Neil Burgess, Colin Lever, and Alastair McClelland for helpful discussions of the manuscript. We acknowledge funding from the ERC ("DEVSPACE" grant) (to F.C.), the BBSRC (grant BB/I021221/1) (to F.C.), the Royal Society (URF fellowship) (to T.W.), and the Swiss Science Foundation (fellowship) (to J.H.).

Received: October 16, 2014
Revised: March 10, 2015
Accepted: April 30, 2015
Published: June 3, 2015

REFERENCES

Bjerknes, T.L., Moser, E.I., and Moser, M.-B. (2014). Representation of geometric borders in the developing rat. Neuron *82*, 71–78.

Blair, M.G., Nguyen, N.N.-Q., Albani, S.H., L'Etoile, M.M., Andrawis, M.M., Owen, L.M., Oliveira, R.F., Johnson, M.W., Purvis, D.L., Sanders, E.M., et al. (2013). Developmental changes in structural and functional properties of hippocampal AMPARs parallels the emergence of deliberative spatial navigation in juvenile rats. J. Neurosci. *33*, 12218–12228.

Brandon, M.P., Bogaard, A.R., Libby, C.P., Connerney, M.A., Gupta, K., and Hasselmo, M.E. (2011). Reduction of theta rhythm dissociates grid cell spatial periodicity from directional tuning. Science *332*, 595–599.

Burak, Y., and Fiete, I.R. (2009). Accurate path integration in continuous attractor network models of grid cells. PLoS Comput. Biol. *5*, e1000291.

Burgess, N., Barry, C., and O'Keefe, J. (2007). An oscillatory interference model of grid cell firing. Hippocampus *17*, 801–812.

Bush, D., and Burgess, N. (2014). A hybrid oscillatory interference/continuous attractor network model of grid cell firing. J. Neurosci. *34*, 5065–5079.

Bush, D., Barry, C., and Burgess, N. (2014). What do grid cells contribute to place cell firing? Trends Neurosci. *37*, 136–145.

Csicsvari, J., Hirase, H., Czurkó, A., Mamiya, A., and Buzsáki, G. (1999). Oscillatory coupling of hippocampal pyramidal cells and interneurons in the behaving Rat. J. Neurosci. *19*, 274–287.

Fuhs, M.C., and Touretzky, D.S. (2006). A spin glass model of path integration in rat medial entorhinal cortex. J. Neurosci. *26*, 4266–4276.

Green, R.J., and Stanton, M.E. (1989). Differential ontogeny of working memory and reference memory in the rat. Behav. Neurosci. *103*, 98–105.

Hafting, T., Fyhn, M., Molden, S., Moser, M.B., and Moser, E.I. (2005). Microstructure of a spatial map in the entorhinal cortex. Nature *436*, 801–806.

Hartley, T., Burgess, N., Lever, C., Cacucci, F., and O'Keefe, J. (2000). Modeling place fields in terms of the cortical inputs to the hippocampus. Hippocampus *10*, 369–379.

Hasselmo, M.E., Giocomo, L.M., and Zilli, E.A. (2007). Grid cell firing may arise from interference of theta frequency membrane potential oscillations in single neurons. Hippocampus *17*, 1252–1271.

Koenig, J., Linder, A.N., Leutgeb, J.K., and Leutgeb, S. (2011). The spatial periodicity of grid cells is not sustained during reduced theta oscillations. Science *332*, 592–595.

Langston, R.F., Ainge, J.A., Couey, J.J., Canto, C.B., Bjerknes, T.L., Witter, M.P., Moser, E.I., and Moser, M.B. (2010). Development of the spatial representation system in the rat. Science *328*, 1576–1580.

Lenck-Santini, P.P., Muller, R.U., Save, E., and Poucet, B. (2002). Relationships between place cell firing fields and navigational decisions by rats. J. Neurosci. *22*, 9035–9047.

Lever, C., Burton, S., Jeewajee, A., O'Keefe, J., and Burgess, N. (2009). Boundary vector cells in the subiculum of the hippocampal formation. J. Neurosci. *29*, 9771–9777.

McNaughton, B.L., Battaglia, F.P., Jensen, O., Moser, E.I., and Moser, M.B. (2006). Path integration and the neural basis of the 'cognitive map'. Nat. Rev. Neurosci. *7*, 663–678.

Monaco, J.D., and Abbott, L.F. (2011). Modular realignment of entorhinal grid cell activity as a basis for hippocampal remapping. J. Neurosci. *31*, 9414–9425.

O'Keefe, J., and Burgess, N. (2005). Dual phase and rate coding in hippocampal place cells: theoretical significance and relationship to entorhinal grid cells. Hippocampus *15*, 853–866.

O'Keefe, J., and Nadel, L. (1978). The hippocampus as a cognitive map (Oxford University Press).

O'Keefe, J., and Speakman, A. (1987). Single unit activity in the rat hippocampus during a spatial memory task. Exp. Brain Res. *68*, 1–27.

Poucet, B., Sargolini, F., Song, E.Y., Hangya, B., Fox, S., and Muller, R.U. (2014). Independence of landmark and self-motion-guided navigation: a different role for grid cells. Philos. Trans. R. Soc. Lond. B Biol. Sci. *369*, 20130370.

Rauch, S.L., and Raskin, L.A. (1984). Cholinergic mediation of spatial memory in the preweanling rat: application of the radial arm maze paradigm. Behav. Neurosci. *98*, 35–43.

Rudy, J.W., Stadler-Morris, S., and Albert, P. (1987). Ontogeny of spatial navigation behaviors in the rat: dissociation of "proximal"- and "distal"-cue-based behaviors. Behav. Neurosci. *101*, 62–73.

Savelli, F., Yoganarasimha, D., and Knierim, J.J. (2008). Influence of boundary removal on the spatial representations of the medial entorhinal cortex. Hippocampus *18*, 1270–1282.

Schenk, F. (1985). Development of place navigation in rats from weaning to puberty. Behav. Neural Biol. *43*, 69–85.

Scott, R.C., Richard, G.R., Holmes, G.L., and Lenck-Santini, P.P. (2011). Maturational dynamics of hippocampal place cells in immature rats. Hippocampus *4*, 347–353.

Skaggs, W.E., McNaughton, B.L., Wilson, M.A., and Barnes, C.A. (1996). Theta phase precession in hippocampal neuronal populations and the compression of temporal sequences. Hippocampus *6*, 149–172.

Solstad, T., Moser, E.I., and Einevoll, G.T. (2006). From grid cells to place cells: a mathematical model. Hippocampus *16*, 1026–1031.

Solstad, T., Boccara, C.N., Kropff, E., Moser, M.B., and Moser, E.I. (2008). Representation of geometric borders in the entorhinal cortex. Science *322*, 1865–1868.

Stewart, S., Jeewajee, A., Wills, T.J., Burgess, N., and Lever, C. (2014). Boundary coding in the rat subiculum. Philos. Trans. R. Soc. Lond. B Biol. Sci. *369*, 20120514.

Taube, J.S., Muller, R.U., and Ranck, J.B., Jr. (1990). Head-direction cells recorded from the postsubiculum in freely moving rats. I. Description and quantitative analysis. J. Neurosci. *10*, 420–435.

Wang, Y., Romani, S., Lustig, B., Leonardo, A., and Pastalkova, E. (2015). Theta sequences are essential for internally generated hippocampal firing fields. Nat. Neurosci. *18*, 282–288.

Wills, T.J., Cacucci, F., Burgess, N., and O'Keefe, J. (2010). Development of the hippocampal cognitive map in preweanling rats. Science *328*, 1573–1576.

Zhang, K., Ginzburg, I., McNaughton, B.L., and Sejnowski, T.J. (1998). Interpreting neuronal population activity by reconstruction: unified framework with application to hippocampal place cells. J. Neurophysiol. *79*, 1017–1044.

Zhang, S.-J., Ye, J., Miao, C., Tsao, A., Cerniauskas, I., Ledergerber, D., Moser, M.-B., and Moser, E.I. (2013). Optogenetic dissection of entorhinal-hippocampal functional connectivity. Science *340*, 1232627.

Zilli, E.A., and Hasselmo, M.E. (2010). Coupled noisy spiking neurons as velocity-controlled oscillators in a model of grid cell spatial firing. J. Neurosci. *30*, 13850–13860.

Neuron

Supplemental Information

A Developmental Switch in Place Cell Accuracy Coincides with Grid Cell Maturation

Laurenz Muessig, Jonas Hauser, Thomas Joseph Wills, and Francesca Cacucci

Figure S1 (related to Figure 1 and Figure 2)

Analysis of Individual Animals

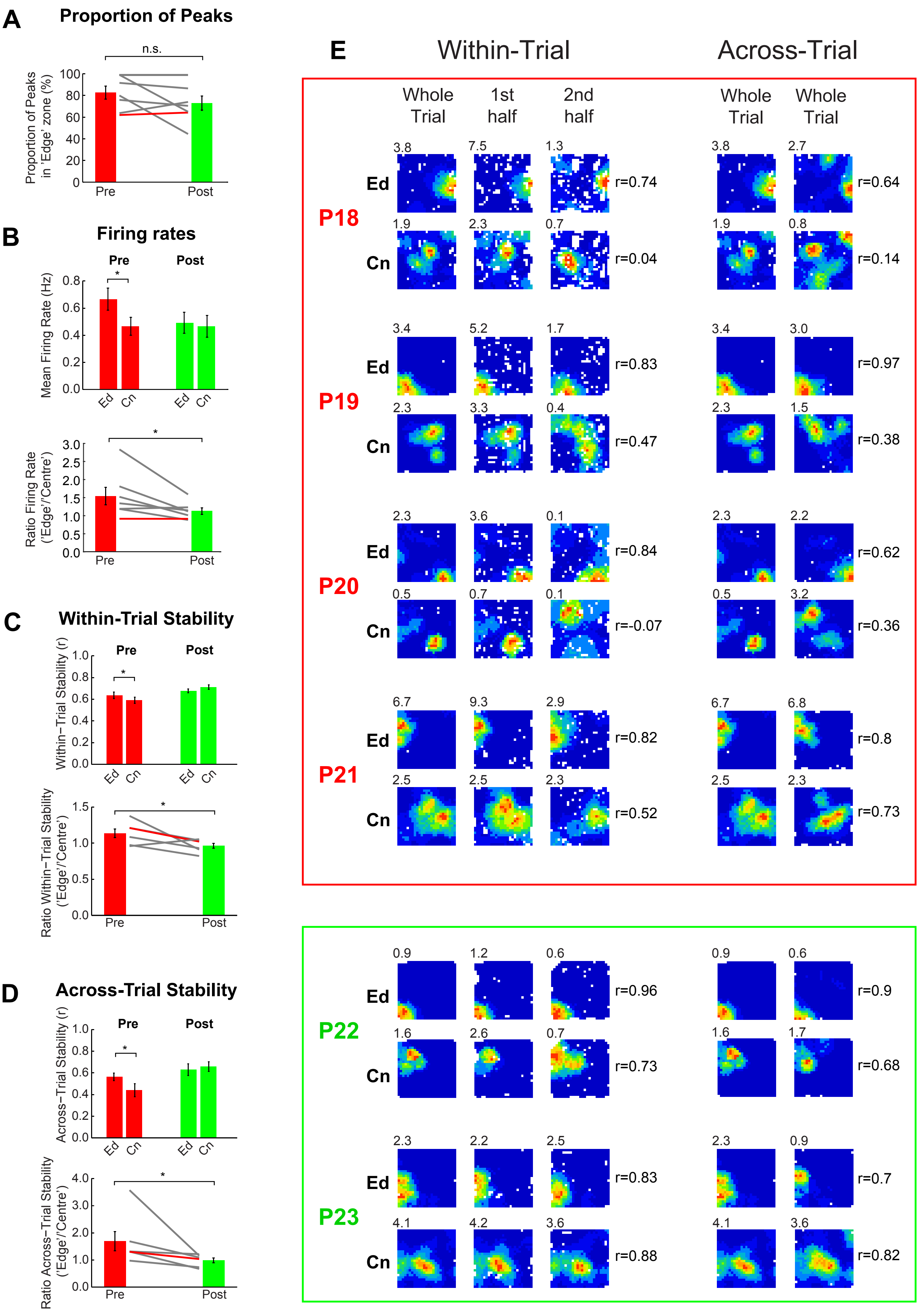

Abrupt Switch Between ‘Edge’ and ‘Centre’ Coding

Distribution of Peaks

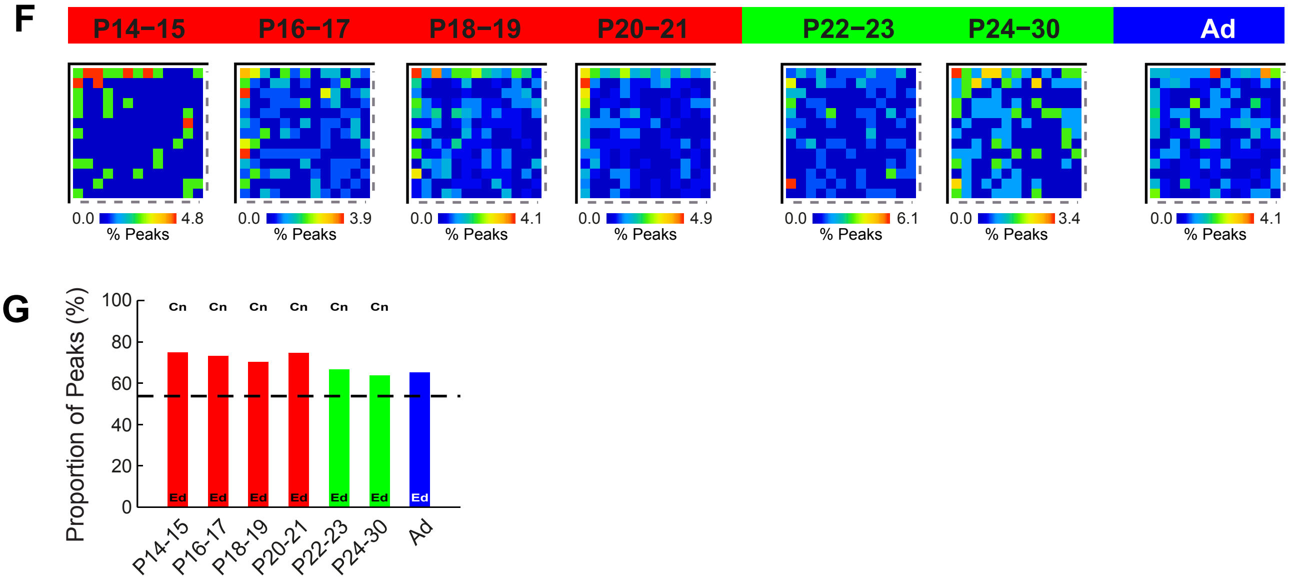

Mean Rate Maps

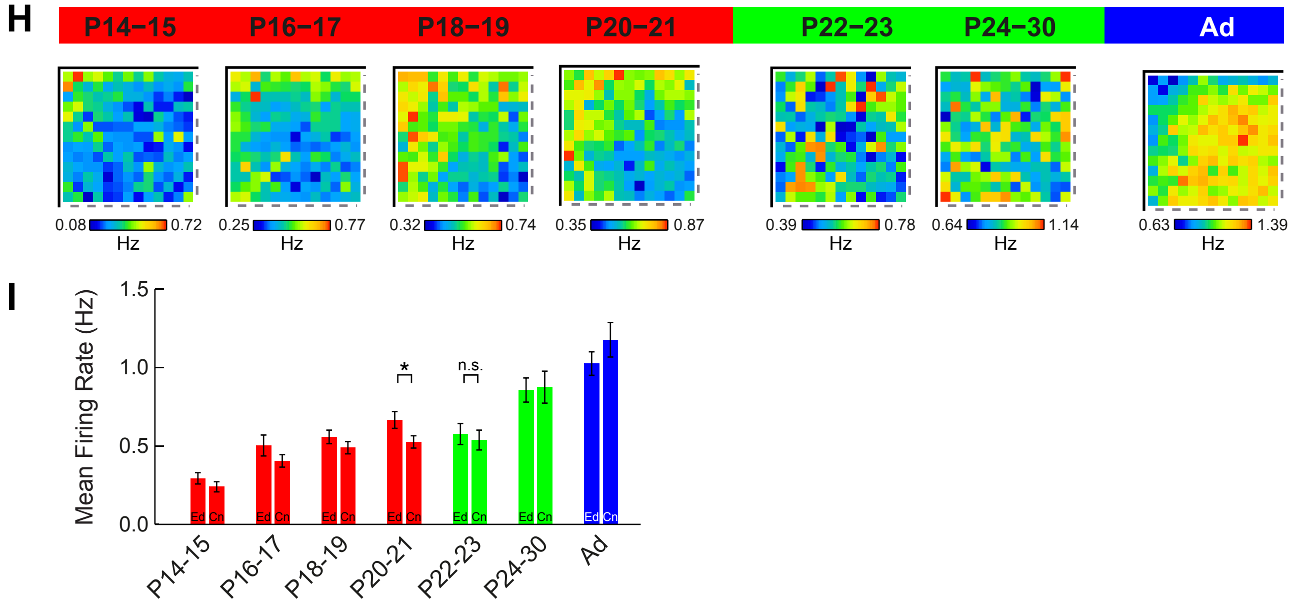

Place Cell Stability in ‘Edge’ versus ‘Centre’ zones

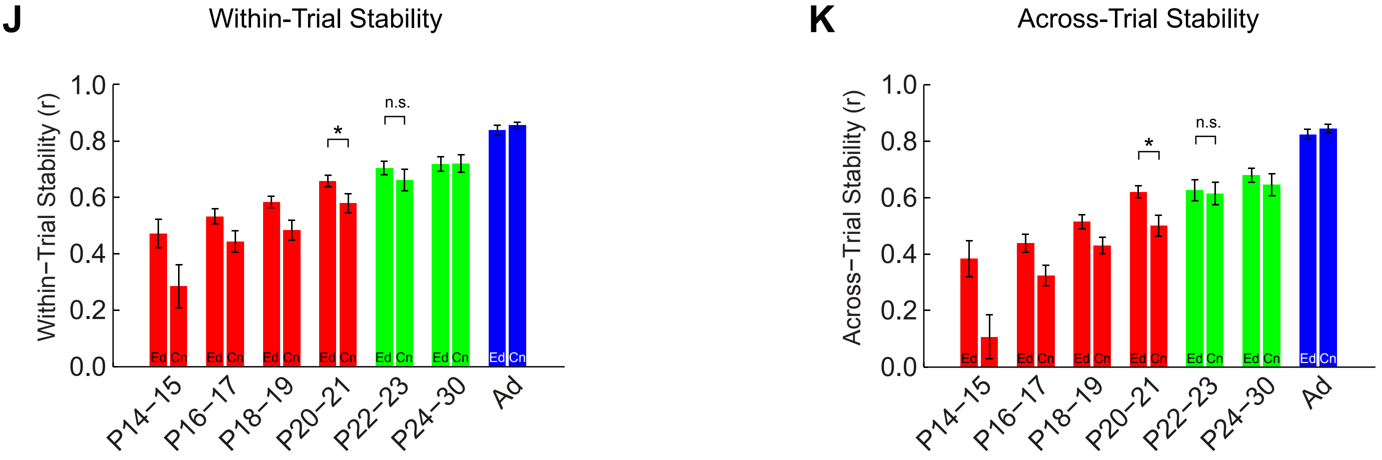

Edge vs centre stability disparity is constant between P16 and weaning age

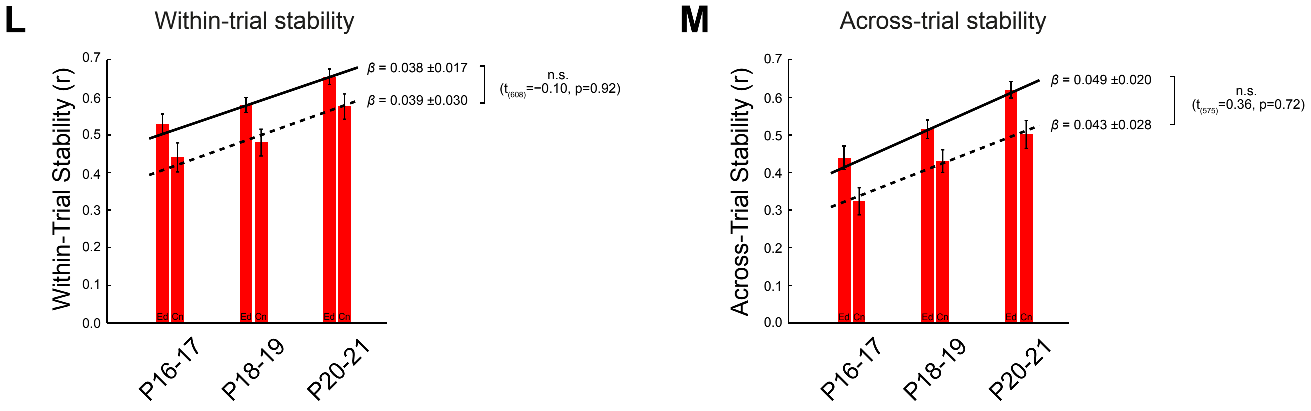

Edge vs centre stability disparity does reduce, abruptly, at weaning age

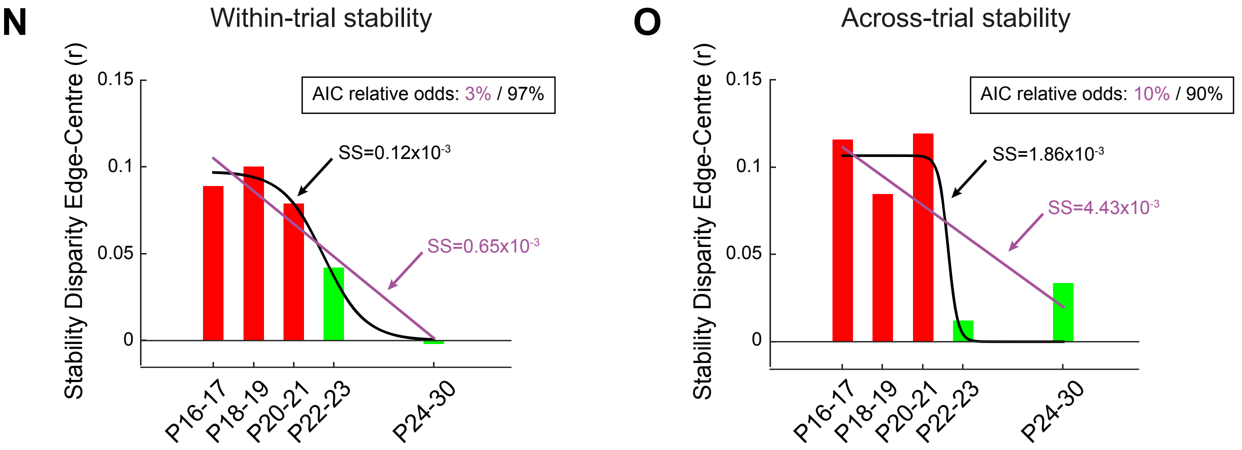

Abrupt emergence of grid cells at weaning age

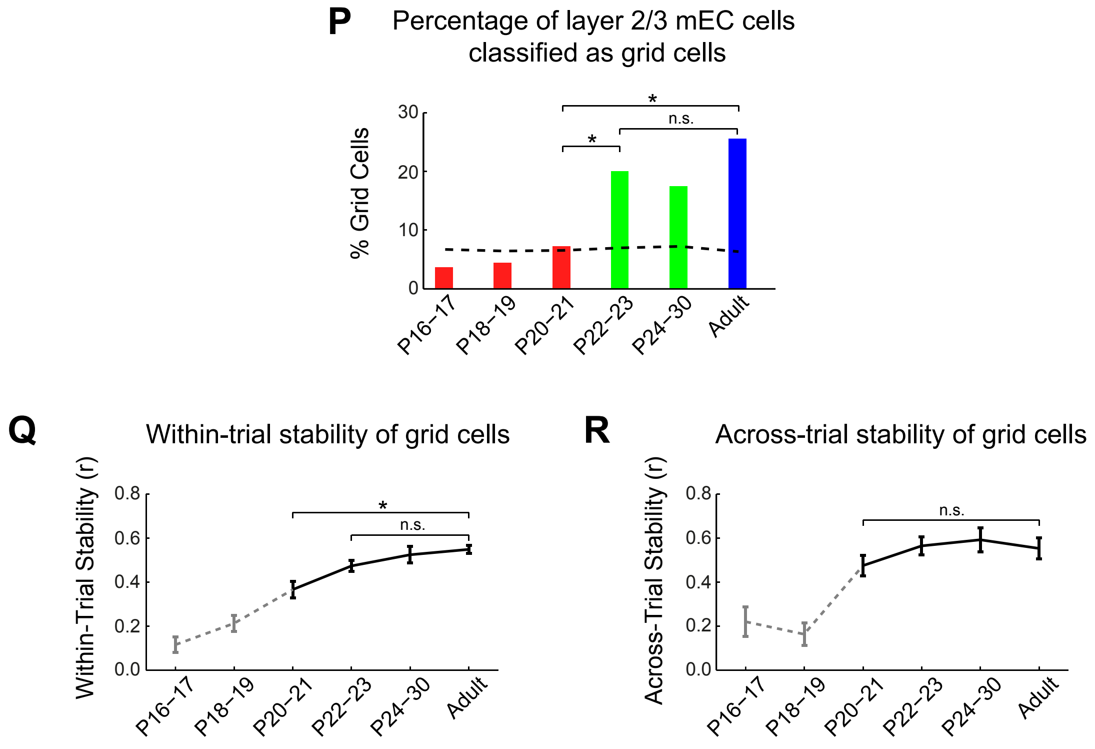

Fig S1: The shift between 'Edge' vs. 'Centre' coding in place cell maps is present in individual animals (A-E) and happens abruptly around weaning (F-O), the same age that a population of grid cells emerges in the superficial layers of the medial entorhinal cortex (P-R). (A-E): Analyses of data obtained from animals where place cells were recorded at least 1 day before and 1 day after weaning. **(A)** Distributions of place field peaks in pre- and post-weanling animals (7 rats). Mean (±SEM) proportion of place field peaks in 'Edge' zone per animal, pre- and post-weaning (Wilcoxon-Signed-Ranks test, p=0.25). Lines represent data from individual animals. Red line corresponds to animal for which example firing rate maps are shown in (E). (**B**) Place cell firing rates are higher in the edge of environment for pre-weanling animals only (7 rats). Top panel: firing rates for 'Edge' (Ed) and 'Centre' (Cn) zones (mean ± SEM; RM-ANOVA: zone*age: $F_{1,12}$=5.3, p=0.030; SME $Zone_{(pre-wean)}$, p=0.002). Bottom panel: Ratios of firing rates between 'Edge' and 'Centre' zones before and after weaning, within each rat (bottom panel; Wilcoxon-Signed-Ranks test, p=0.043). Key as in (A). * denotes significance at p<0.05. (**C**) Place fields are less stable within-trial in environment centre in pre-weanling animals only (6 rats, as one rat included in A,B had no field peaks in the centre of the environment (see S1A), hence edge versus centre comparisons are not possible). Top panel: Within-trial stability of place cells with peak firing locations in the 'Edge' (Ed) and 'Centre' (Cn) zones of the environment (mean correlation ±SEM; RM-ANOVA: zone*age: $F_{1,10}$=5.2, p=0.048; SME $Zone_{(pre-wean)}$, p=0.045). Bottom panel: Ratios of within-trial stability between 'Edge' and 'Centre' zones before and after weaning, within each rat (Wilcoxon-Signed-Ranks test, p=0.046). Key as in (A). * denotes significance at p<0.05. (**D**) Place fields are less stable across-trial in environment centre in pre-weanling animals only (6 rats). Top panel: Across-trial stability of place cells with peak firing locations in the 'Edge' (Ed) and 'Centre' (Cn) zones of the environment (mean correlation ±SEM; RM-ANOVA: zone*age: $F_{1,10}$=5.1; p=0.047, SME $Zone_{(pre-wean)}$, p=0.016). Bottom panel: Ratios of across-trial stability between 'Edge' and 'Centre' zones before and after weaning, within each rat (Wilcoxon-Signed-Ranks test, p=0.028). Key as in A. * denotes significance at p<0.05 (**E**) False colour firing rate maps for example cells recorded from one animal between P18-23. Within each age group, maps from one cell with peak in Edge (Ed, top) and one with peak in 'Centre' (Cn, bottom) of environment are shown. Leftmost maps ("Within-trial") show whole recording session (first column), first half of session (2nd column) and second half of session (3rd column). Rightmost maps ("Across-trial") show two adjacent recording trials. Numbers top left of maps are peak firing rates (Hz). R-values for within and across trial correlations are shown next to within and across trial maps, respectively. **(F-G) Place cell firing is more concentrated close to environment boundaries in pre-weanling (P14-P21) than in post-weanling (P22-P30) and adult rats. (F)** Distribution of place cell peaks (%) in recording environment shown, at fine timescale, as false-colour quadrant mean maps (corresponding to fig 1B). (**G**) Proportion of place cell peaks in 'Edge' (Ed – bottom portion of each bar) and 'Centre' (Cn – top portion of each bar) zone of the environment at fine timescale (corresponding to fig 1C). Black dashed line indicates value for an even distribution of peaks across zones. The proportion of peaks in the edge zone is significantly greater than the adult proportion at P20-21, but not at P22-23 (Uncorrected p-values from Z-test versus adult proportion: P14-15: Z=1.31, p=0.18; P16-17: Z=2.03, p=0.043; P18-19: Z=1.59, p=0.11; P20-21: Z=2.92, p=0.004; P22-23: Z=0.4, p=0.69; P24-30: Z=-0.22, p=0.83). **(H)** Distribution of place cell firing rates shown, at fine timescale, as quadrant mean rate maps of overall, unsmoothed, firing rate (in Hz) for all recorded place cells in each age group (corresponding to fig 1D). **(I)** Mean place cell firing rate (±SEM) in 'Edge' (Ed) vs. 'Centre' (Cn) zones of environment at fine timescale (corresponding to fig. 1E). There is a significant difference between overall mean rate in the edge and centre zones at P20-21, but not at P22-23 (p-values from uncorrected paired t-tests, full list; P14-15: t=1.96, p=0.057; P16-17: t=1.95, p=0.053; P18-19: t=2.22, p=0.03; P20-21: t=3.58, p<0.001; P22-23: t=0.91, p=0.36; P24-30: t=0.26, p=0.8; adult: t=1.73, p=0.085).**(J-K) Place cell firing is more stable in the 'edge' zone then the 'centre' zone of the environment in pre-weanling (P14-P21), but not post-weanling (P22-P30) and adult rats. (J)** Within-trial stability (mean correlation ±SEM) shown at fine timescale (corresponding to fig. 2A). There is a significant difference between edge and centre stability at P20-21, but not at P22-23 (p-values from uncorrected independent samples t-tests, full list of values; P14-15: t=2.02, p=0.051; P16-17: t=1.9, p=0.06; P18-19: t=2.63, p=0.009; P20-21: t=2.59, p=0.01; P22-23: t=0.97, p=0.34; P24-30: t=0.25, p=0.8; adult: t=0.22, p=0.83). (**K**) Across-trial stability (mean correlation ±SEM) shown at fine timescale (corresponding to fig. 2D). There is a significant difference between edge and centre stability at P20-21, but not at P22-23 (p-values from uncorrected independent samples t-tests, full list of values; P14-15: t=2.75, p=0.01; P16-17: t=2.51, p=0.01; P18-19: t=2.68, p=0.008; P20-21: t=3.07, p=0.003; P22-23: t=0.4, p=0.69; P24-30: t=0.52, p=0.6; adult: t=0.3, p=0.77). **(L-M) The disparity in stability between place cells in the 'edge' and 'centre' zones is constant from P16, until weaning age.** Linear regression was performed on

stability versus age, separately for all ‘edge’ and all ‘centre’ cells recorded between P16 and P21. Solid black lines show the best fit for ‘edge’ place cells, black dashed lines show the best fit for ‘centre’ place cells. Red bars show the age bin means and SEMs for P16 – P21 (same data as S1J, K), for reference. The slopes for the ‘edge’ and ‘centre’ regressions are extremely similar (slope constants β (±95% CI) and statistical significance for slope difference shown on graphs, see also supplemental methods), demonstrating that the reduction of stability in the centre of the environment, relative to the edge, does not change before P22, when grid cells emerge. The youngest rats (P14-15) were not included, as these animals do show an additional sharp change in stability (see S1J,K). This change is likely due to vision onset (median eye-opening age = P15), which marks a significant developmental discontinuity, both in terms of sensory inputs available to the hippocampus, and for the development of the head-direction cell system (Tan et al., 2015). **(N-O) The disparity in stability between place cells in the ‘edge’ and ‘centre’ zones reduces abruptly at weaning age.** Red and green bars show the disparity between the mean stability of ‘edge’ place cells and the mean stability of ‘centre’ place cells at age bins P16 – P30 (c.f. figure S1J,K). The black lines show the best fits of a logistic function to these data, the magenta lines show the best fits of a straight line (to equate the number of free parameters between the fits, the logistic function was constrained by our prior hypothesis such that (1) the inflexion point was at weaning age, (2) the difference between ‘edge’ and ‘centre’ stability for post-weanling data was zero, see supplemental methods for details). The Sum of Squares of the residuals (‘SS’) for these fits are shown on the graphs: in both cases, the logistic function has a lower SS than the straight line. We used the Akaike Information Criterion (AIC; see supplemental methods) to assess the relative likelihood of the logistic and straight line fits being correct (see ‘AIC relative odds’ on graphs), in both cases the logistic function was more likely (relative odds: within-trial, 3% vs 97%, across-trial, 10% vs 90%). The improvement of stability in the centre of the environment relative to the edge between P16 and P30 is therefore much better modelled by an abrupt process centred at weaning than a gradual process occurring throughout this period. **(P-R) Abrupt emergence of grid cells at weaning age. (P)** Percentage of superficial mEC cells classified as grid cells across development. Grid cells were defined following (Wills et al., 2010) but using the spatial binning and smoothing parameters used in the current study. The black dashed line shows the 95% confidence level for the percentage of grid cells expected in spatially randomised data. The percentage of grid cells at P20-21 is significantly different to that of P22-23 and that of adults (2-sample Z-test: P20-21 versus P22-23, Z=11.8, p<0.001; P20-21 versus Ad, Z=15.2, p<0.001) but the percentage of grid cells at P22-23 is not significantly different to that of adults (Z=1.01, p=0.31). **(Q, R)** Mean stability of superficial mEC grid cells (±SEM) across ages. Across-trial stability is not significantly different from adult by P20, within-trial stability by P22 (Tukey HSD, sig. threshold p=0.05). Lines are greyed-out between P16-P19 as the percentage of grid cells is less than expected in spatially randomised data: cells classified as grids at these ages are treated as false-positives. Panels (P-R) are a re-analysis of previously published data (Wills et al., 2010).

Figure S2 (related to Figure 1 and Figure 2)

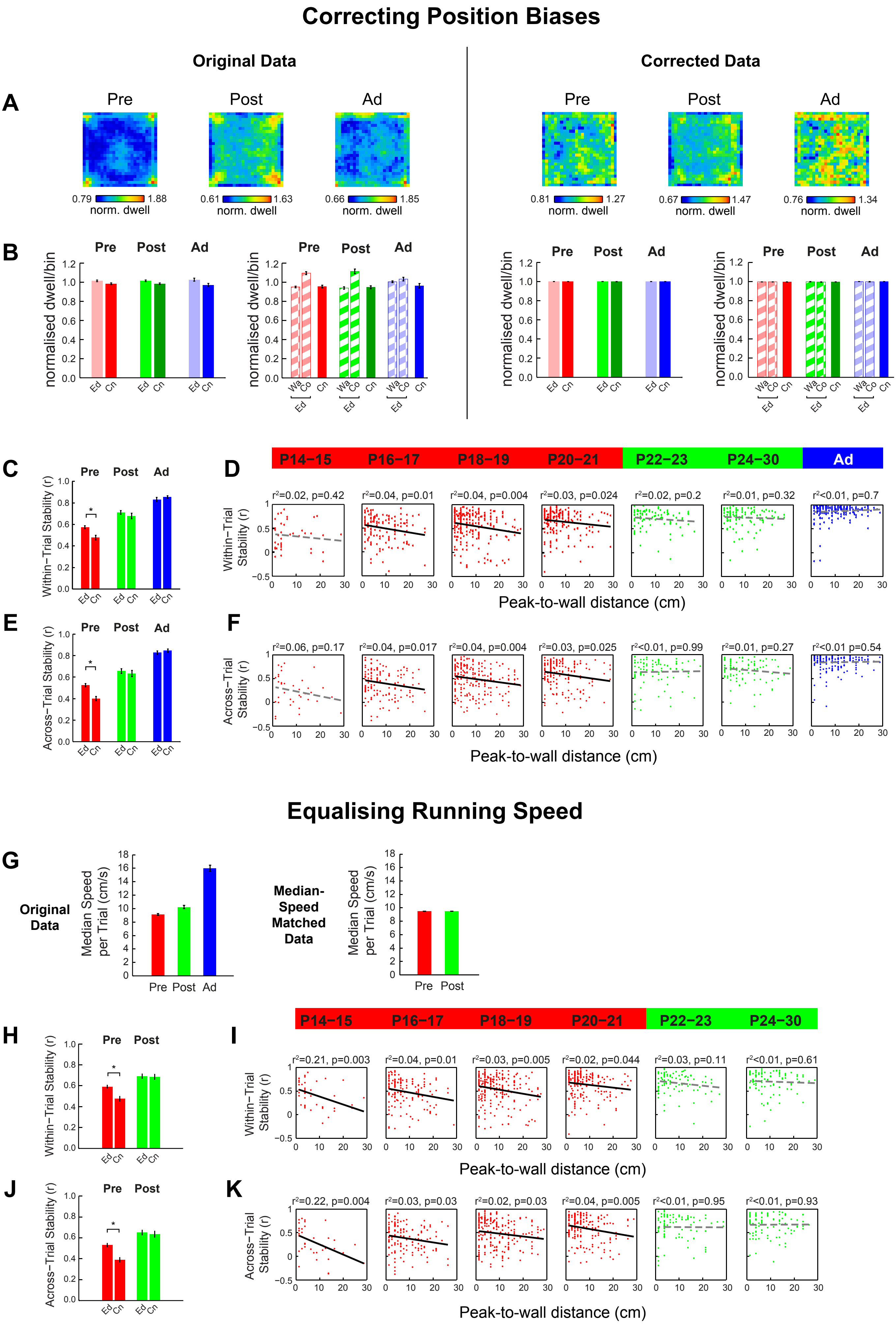

Equalising Firing Rates by Age

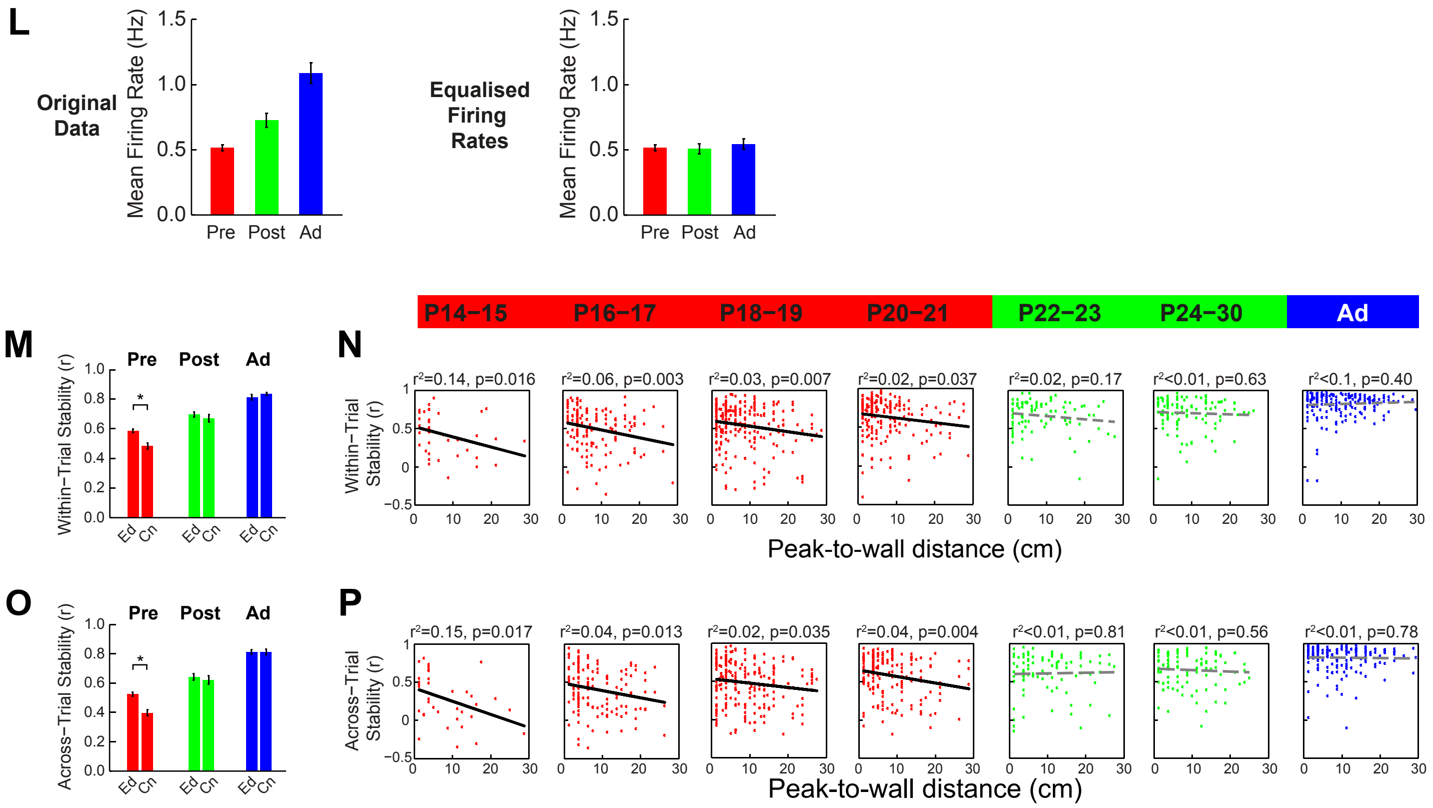

Equalising Firing Rates Across Zones

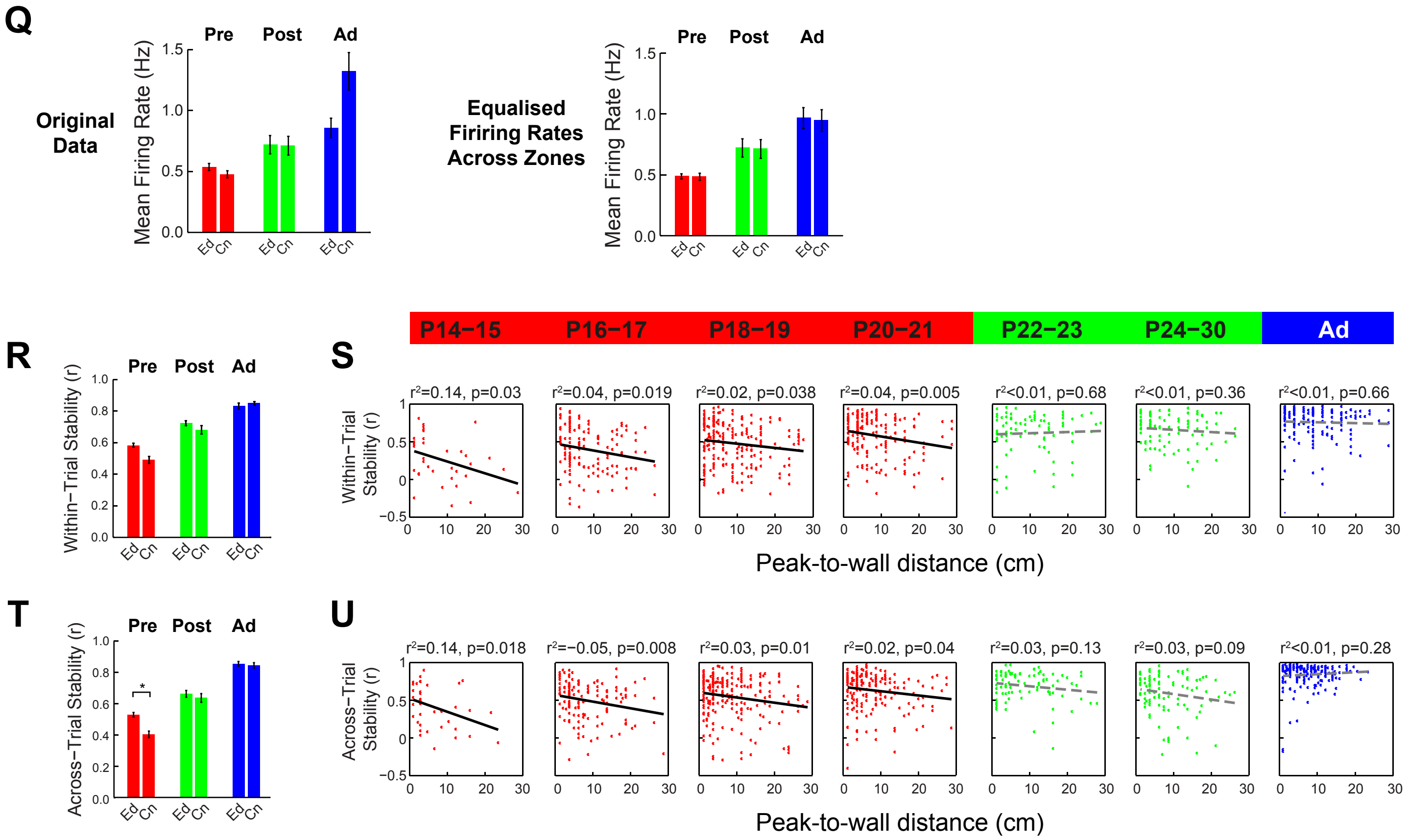

Fig S2: Increased stability of place fields near walls in pre-weanling rats is not related to developmental changes in behaviour or firing rate. (A-F) Place fields are less stable in the centre of the environment in pre-weanling animals, after correcting position sampling biases. (A) False colour maps of mean normalised dwell times for pre-weanling (Pre), post-weanling (Post) and adult (Ad) animals before (left) and after (right) correction of position biases. A normalised dwell of one represents an environment sampled with an equal dwell time in every spatial bin. **(B)** Far left: bar chart depicting mean normalised dwell per bin in the 'Edge' (Ed) and 'Centre' (Cn) zones (±SEM). At all ages, there is a slight bias for greater dwell time in the 'Edge' zone. Second-from-left: mean normalised dwell per bin (±SEM), after sub-dividing the 'Edge' zone into 'Corner' (Co) and 'Wall' (Wa) (see supplemental methods). Rat pups show higher dwell per bin in the corners of the environment, compared to adults (see also (A)). Second-from-right, far right: mean normalised dwell per bin (±SEM) after correction of position biases by down-sampling of data (see supplemental methods for details). **(C)** Within-trial stability of place cells with peak firing locations in the 'Edge' (Ed) and 'Centre' (Cn) zones of the environment, after position biases correction (corresponding to fig. 2A, ANOVA: zone*age: $F_{2,1005}$=3.1, p=0.048; SME $Zone_{(pre-wean)}$, p<0.001). **(D)** Scatter plots of within-trial stability versus distance from peak to nearest wall, after position biases correction (corresponding to fig. 2C). Solid black lines represent linear regression lines of best fit and are significant at p<0.05 level, r^2 and p for regression are indicated above plots. **(E)** Same as (C) but for across-trial stability (corresponding to fig. 2D, F, ANOVA: zone*age: $F_{2,943}$=4.6, p=0.01; SME $Zone_{(pre-wean)}$, p<0.001). **(F)** Same as (D) but for across-trial stability. (**G-K**) **Place fields are less stable in the centre of the environment in pre-weanling animals, after equalising running speed across development. (G)** Left, bar chart depicting median speed per trial (±SEM) of pre-weanling (Pre), post-weanling (Post) and adult (Ad) animals as per original data. Right, bar chart depicting median speed per trial (±SEM) of pre-weanling (Pre) and post-weanling (Post) animals after equalising running speed across these groups (adults not included in analysis, see supplemental methods for details). (**H**): Within-trial stability of place cells with peak firing locations in the 'Edge' (Ed) and 'Centre' (Cn) zones of the environment, after equalising running speed (corresponding to fig. 2A, ANOVA: zone*age: $F_{1,808}$=4.5, p=0.035; SME $Zone_{(pre-wean)}$, p<0.001). (**I**): Scatter plots of within-trial stability versus distance from peak to nearest wall after equalising running speed (corresponding to fig. 2C). Key for plots as in (F). **(J)** Same as (H) but for across-trial stability (corresponding to fig. 2D, F; ANOVA: zone*age: $F_{1,767}$=6, p=0.015; SME $Zone_{(pre-wean)}$, p<0.001). **(K)** Same as (I) but for across-trial stability. (**L-P) Place fields are less stable in the centre of the environment in pre-weanling animals, after equalising firing rates across ages. (L)** Mean firing rates of place cells (±SEM) recorded from pre-weanling (Pre), post-weanling (Post) and adult (Ad) animals before (left) and after (right) firing rate equalisation procedure (see supplemental methods for details). **(M)** Stability within recording trials (mean correlation ±SEM) across age groups in different zones ('Ed' and 'Cn') after firing rates equalisation (corresponding to fig. 2A, ANOVA: zone*age: $F_{2,1005}$=3.4, p=0.033; SME $Zone_{(pre-wean)}$, p<0.001). **(N)** Scatter plots of within-trial stability versus distance from peak to nearest wall after firing rate equalisation (corresponding to fig. 2C). Key for plots as in F. **(O)** Same as (M) but for across-trial stability (corresponding to fig. 2D, F; ANOVA: zone*age: $F_{2,943}$=4.2, p=0.015; SME $Zone_{(pre-wean)}$, p<0.001). **(P)** Same as (N) but for across-trial stability. **(Q-U) Place cell stability, across trials, is lower in the centre of the environment in pre-weanling animals, after equalising firing rates across environment edge and centre zone. (Q)** Mean firing for place cells with peaks in the 'Edge' (Ed) and 'Centre' (Cn) zones of the environment (±SEM) before (left) and after (right) equalisation of firing rates across zones (see supplemental methods for details). **(R)** Within-trial stability of place cells with peak firing locations in the 'Edge' (Ed) and 'Centre' (Cn) zones of the environment, after equalising firing rates across zones (corresponding to fig. 2A). After equalisation, there is no longer a significant interaction between age and zone (ANOVA: zone*age: $F_{2,971}$=2.1, p=0.12). **(S)** Scatter plots of within-trial stability versus distance from peak to nearest wall after equalising firing rates across zones (corresponding to fig. 2C). Key for plots as in (F). **(T)** Same as (R), but for across-trial stability (corresponding to fig. 2D, F; ANOVA: zone*age: $F_{2,911}$=3.4, p=0.036; SME $Zone_{(pre-wean)}$, p<0.001). **(U)** Same as (S), but for across-trial stability.

* denotes significance at p<0.05

Figure S3 (related to Figure 3)

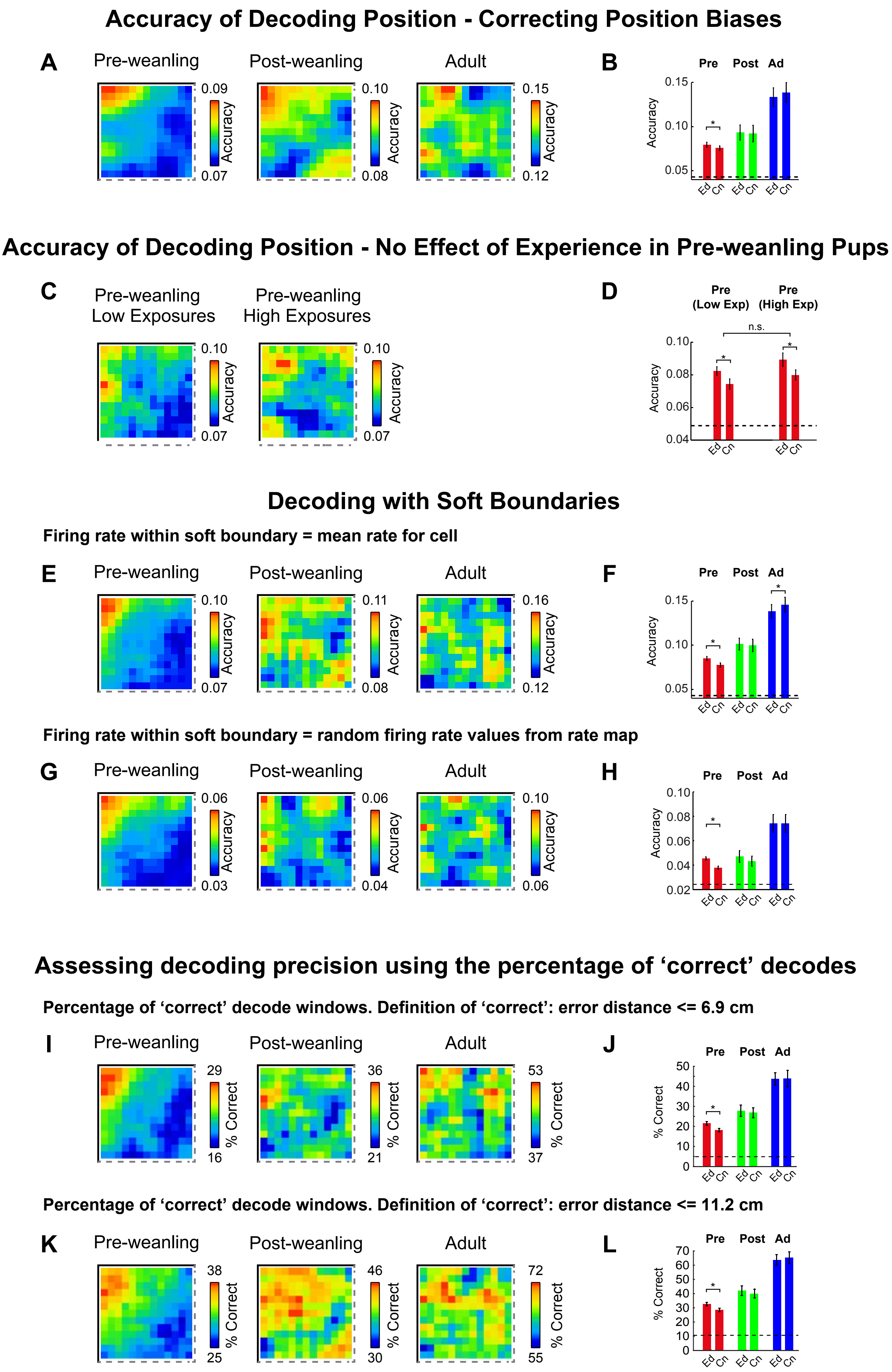

Fig S3: Supplemental Bayesian position decoding analyses. (A, B) Correcting for position biases across development does not affect the differences in decoding accuracy across age groups. Data contributing to the Bayesian decoding analysis was down-sampled such as to equalise the mean dwell time per bin between the 'Edge' and 'Centre' zones (see figure S2A and B; supplemental methods for details of dwell bias and data down-sampling). (**A**) False colour quadrant mean maps of reconstruction accuracy for each age group after correcting position biases (corresponding to fig. 3C). (**B**) Mean accuracy in 'Edge' (Ed) and 'Centre' (Cn) zones of the environment (±SEM). Black dashed line indicates mean expected accuracy from decoding spatially shuffled data. Reconstruction accuracy is higher near to boundaries in pre-weanling, but not in post-weanling or adult rats (corresponding to fig. 3D; ANOVA, Age*Zone, $F_{2,73}$=4.2, p=0.019, SME $Zone_{(pre\text{-}wean)}$ p<0.001, SME $Zone_{(post\text{-}wean)}$, p=0.62, SME $Zone_{(adult)}$, p=0.07). **(C, D) Increased decoding accuracy close to walls in pre-weanling pups is not affected by the number of previous exposures to the recording environment.** The data shown are filtered by whether the rat had either a low or a high number of previous exposures to the environment when the ensemble was recorded (low: 2 – 4 previous exposures, 15 ensembles, median previous exposures = 3; high: 14-26 previous exposures, 14 ensembles, median previous exposures = 18; 2 and 26 represent the absolute minimum and maximum number of previous exposures for the entire pre-weanling dataset). (**C**) False colour quadrant mean maps of reconstruction accuracy for pre-weanling pups, for low or high numbers of previous exposures to the environment. (**D**) Mean accuracy (±SEM) in 'Edge' (Ed) and 'Centre' (Cn) zones of the environment. Black dashed line indicates mean expected accuracy from decoding spatially shuffled data. Reconstruction accuracy is higher near to boundaries for both the low and high exposures groups, but there is no significant effect of the number of exposures (ANOVA, Exp*Zone: Zone, $F_{1,27}$=22, p<0.001; Exp, $F_{1,27}$=2.2, p=0.15; Exp*Zone, $F_{1,27}$=0.14, p=0.72. SME $Zone_{(Low\ Exp)}$, p=0.002, SME $Zone_{(High\ Exp)}$, p=0.004). (**E**-**H**) **Decoding with 'soft boundaries' does not affect accuracy differences between zones across age groups (see supplemental methods for details).** The position decoding analysis was run with 'soft boundaries', whereby the actual environment was embedded into a larger space, into which position could potentially be decoded (the 'soft boundary'). (E, F) and (G, H) differ with respect to the value of the firing rate ascribed to complex spike cells in the soft boundary region. (**E, F**) For each complex spike cell, firing rates ($f_i(x)$) within the soft boundary region were set to the overall mean firing rate for the cell. (**E**) False-colour quadrant mean maps of reconstruction accuracy for each age group (corresponding to fig. 3C). (**F**) Mean accuracy in 'Edge' ('Ed') and 'Centre' ('Cn') zones of the environment (±SEM). Reconstruction accuracy is higher near boundaries in pre-weanling, but not in post-weanling or adult rats (corresponding to fig. 3D; ANOVA Age*Zone, $F_{2,73}$=8.6, p<0.001, SME $Zone_{(pre\text{-}wean)}$ p<0.001, SME $Zone_{(post\text{-}wean)}$, p=0.63, SME $Zone_{(adult)}$, p=0.03). (**G**, **H**) Same as (E, F) but with firing rates for each complex spike cell ($f_i(x)$) in the soft boundary region set to a selection of rate values randomly chosen from the corresponding firing rate map of the actual environment. Note that overall accuracy is reduced, however, the specific increase in accuracy in the 'Edge' zone in pre-weanling rats is preserved (ANOVA, Age*Zone, $F_{2,73}$=3.5, p=0.035, SME $Zone_{(pre\text{-}wean)}$ p<0.001, SME $Zone_{(post\text{-}wean)}$, p=0.16, SME $Zone_{(adult)}$, p=0.99). **(I-L) Assessing decoder precision using the percentage of 'correct' decode windows yields comparable results to accuracy.** The estimate of position in each decode window was classified as 'correct' if the error distance fell below a certain threshold. **(I, J)** Threshold for a correct decode defined as the median adult error distance (6.9cm). **(I)** False-colour quadrant mean maps of the percentage of correct decodes for each age group (corresponding to fig. 3C). **(J)** Mean percentage of correct decodes in 'Edge' ('Ed') and 'Centre' ('Cn') zones of the environment (±SEM). As observed for decoding accuracy, the percentage of correct decode windows is greater in the 'Edge' zone than in the 'Centre' zone, in pre-weanling rats only (corresponding to fig. 3D; ANOVA, Age*Zone, $F_{2,73}$=3.2, p=0.046, SME $Zone_{(pre\text{-}wean)}$ p<0.001, SME $Zone_{(post\text{-}wean)}$, p=0.50, SME $Zone_{(adult)}$, p=0.91). **(K, L)** Same as (I, J) but using the mean adult error distance (11.2cm) as threshold for correct decodes. The percentage of correct decode windows is greater in the 'Edge' zone than in the 'Centre' zone, in pre-weanling rats only (corresponding to fig. 3D; ANOVA Age*Zone, $F_{2,73}$=4.7, p=0.012, SME $Zone_{(pre\text{-}wean)}$ p<0.001, SME $Zone_{(post\text{-}wean)}$, p=0.23, SME $Zone_{(adult)}$, p=0.39).

Supplemental Methods

Analysis of place field position and stability in individual animals. A subset of rats (n=7) contained at least 1 day of recording data both before and after weaning (P21). Data from these animals were analysed in order to assess whether the developmental changes seen in the entire place cell sample were also visible in individual animals. To assess changes in the position of place field peaks, the proportion of peaks in the edge zone was compared pre- and post-weaning, within animal (figure S1A; Wilcoxon signed test). To assess the distribution of place cell firing, the overall mean firing rates for each animal, in the edge and centre, and pre- and post-weaning, were analysed using a repeated measures ANOVA (figure S1B top panel). To further assess trends within animal, the pre- and post-weaning ratios of firing rates in the edge versus the centre were compared (figure S1B bottom panel). To assess trends in stability of place cell firing, a repeated-measures ANOVA was run on the mean stability within each animal (within and across trials) across the two zones of the environment. Additionally we calculated the ratios of correlation coefficients (Z-transformed) between 'Edge' and 'Centre' before and after weaning for each animal. Average ratios before and after weaning were compared with a Wilcoxon-Signed-Ranks test.

Linear regression of age versus stability for edge and centre cells. To assess and compare developmental trends in stability for cells in the 'edge' and 'centre' zones, separate linear regressions were performed for each zone, were the independent variable was defined as the age of the rat (in post-natal days) and the dependent variable was the stability (either across- or within-trial) of the cell. The difference between the 'edge' and 'centre' regression slopes was tested by a t-test, were t was defined as:

$$t = \frac{\beta_1 - \beta_2}{SE_{b1-b2}}$$

where β_1 and β_2 are the slopes of the 'edge' and 'centre' regression coefficients, SE_{b1-b2} is the standard error of the difference between the coefficients, and the degrees of freedom are equal to $n_1 + n_2 - 4$, where n_1 and n_2 are the numbers of cells in the edge and centre regressions, respectively. See (Zar, 2010) for further details. The same method was used to compare the slopes of the stability versus distance-to-wall regressions, see main manuscript figure 2.

Line-fitting the changes in edge-centre stability disparity over age. To further investigate the developmental trends in the disparity between edge and centre stability, we tested whether changes in disparity were better described by functions modelling either a gradual, or an abrupt change. To do this, we found the best fit to the data (that which minimised the sum of the squared Y-residuals) for two functions:

(1) A straight line function, describing a gradual change over age:

$$Y = A + BX$$

where B represents the slope of the line, and A represents the intercept between the line and the line defined by x=0 (i.e. equivalent to linear regression).

(2) A logistic function, which describes an abrupt or sigmoid change over age:

$$Y = A + \frac{(B - A)}{1 + e^{-k(X_0 - X)}}$$

where B represents the upper asymptote of the curve (difference pre-weaning), A represents the lower asymptote of the curve (difference post-weaning), X_0 represents the inflexion point of the curve, and k represents the steepness of the curve. To equate the number of free parameters between the functions, two of the four variables defining the logistic function were constrained by our prior hypothesis to fixed values: the lower asymptote of the curve, A, was set to zero (equivalent to no difference between edge and centre stability after weaning); and the inflexion point of the curve, X_0, was set to weaning age, reflecting our hypothesis that any changes in place cell stability are related to grid cell emergence. The best fit to the data for the parameters k and B (the steepness of the change, and the stability disparity for pre-weanling animals, respectively) were then defined iteratively (using the Matlab NLINFIT function).

For both functions, Y is defined as the difference between the mean 'edge' stability and the mean 'centre' stability in that age bin (for both within- and across-trial stability), and X is defined as age (mid-point of age bin). The fit of these two functions was then compared by examining the sum of squares of the Y-residuals (SS): for both within- and across-trial stability, the SS was smaller for the logistic fit than the linear fit, indicating that the data is better described by an abrupt rather than a linear developmental process. To further quantify the extent to which the logistic function was a better fit to the data, we used the Akaike Information Criterion (AIC), an information theory-based measure which can be used to estimate the relative goodness-of-fit of two models, see (Burnham and Anderson, 2002) for further details. The AIC (corrected for small samples) for each line fitting was defined as:

$$AIC = N.\ln\left(\frac{SS}{N}\right) + 2K + \frac{2K(K+1)}{N - K - 1}$$

Where N is the number of data points, SS is the sum of the squared residuals and K is the number of parameters in the model, plus one (as the error in the residuals counts as a model parameter). A smaller AIC corresponds to a better fit. Then, the relative probability, P, that the model with the larger SS is the correct model for the data, relative to the model with the smaller SS, is defined as:

$$P = \frac{e^{-0.5\Delta}}{1 + e^{-0.5\Delta}}$$

Where Δ is the difference between the AICs for the model with the larger SS, and the model with the smaller SS, respectively.

Equalising dwell time between the edge and centre of the environment. Data was sub-sampled so as to equalise the mean dwell times per spatial bin in the edge (distance to nearest wall <= 10cm) and centre (distance to nearest wall > 10cm) zones of the environment. As inspection of the mean dwell time maps showed that the prevalent position bias was for rats to spend more time in the corners of the environment (see figure S2A), for the purposes of sub-sampling, the 'edge' zone was further sub-divided into 'corner' (distances to two nearest walls were both <= 10cm) and 'wall' (distance to the nearest wall <= 10cm, but the distance to the second nearest wall > 10cm) zones. Mean dwell time per spatial bin was equalised between 'centre', 'corner' and 'wall', which also resulted in an equalisation of dwell time between the 'centre' and 'edge' zones used for all main analyses.

For each recording trial that contributed to the analysis, the dwell time per spatial bin in each zone was equalised using the following method: the mean dwell time per spatial bin was calculated for each zone, and the lowest value of these was set as the target dwell per bin for the remaining two zones. In each of the two zones to be sub-sampled, the spatial bins were sorted on the basis of dwell time, and, starting with the bin with the greatest dwell time, a random selection of position samples were removed, so as to match the total dwell time in that spatial bin to the target dwell per bin for the trial. This process was repeated until the *overall* dwell per bin in the zone matched the target dwell per bin for the trial. Spikes occurring within the duration of the discarded position samples were also removed from the data.

For both position sub-sampling analyses presented (Figures S2C-F and S3A,B), the randomised sub-sampling was repeated 100 times, and scores of place cell stability, place cell peak-to-wall distance (S2C-F) and complex-spike ensemble decoding accuracy (S3A,B) were calculated for each repeat. For figure S2C and S2E, the final ANOVA and regressions were performed using the median stability and peak-to-wall distance for each place cell, across the 100 repeated sub-samples. For figure S3B, the final ANOVA was performed on the median accuracy for each ensemble and for each zone ('Edge' versus 'Centre') across the 100 repeated sub-samples. In all cases, the population of cells contributing to the sub-sampled analysis were the same as those for the main analyses. For figure S3A-B, position sub-sampling was applied to the training data for the Bayesian decoder, such that the estimates of probability of being in a given spatial bin ($\mathrm{P}(x)$) and the mean firing rate in each spatial bin ($f_i(x)$) was calculated using data in which dwell time per bin was equalised between the edge, corner and centre zones.

Equalising running speeds between pre- and post-weanling rats. To control for the effect of running speed variation across ages, developmental trends in place cell stability were also analysed after data was filtered so as to match median speed across all pre- and post-weanling animals. The mean of the trial median speeds for all pup data, recorded between P14 and P30, after immobility filtering (speed < 2.5cm/s), was 9.51cm/s. For each individual trial, position and spike data were filtered such that a) all immobility was excluded, b) the median of the remaining speeds was equal

to 9.51cm/s. Thus, for each trial, speed limits s1 > 2.5 cm/s and s2 <∞ were chosen such that the trial median was equal to 9.51cm/s. Spike and position data outside this range were discarded, rate maps were constructed, and analyses of place cell stability were then applied. Adult data were excluded from this control analysis, due to an excessive discrepancy between pup and adult running speeds (see figure S2G).

Equalising place cell firing rates between age groups. To match mean place cell firing rates between pre-weanling, post-weanling and adult groups, the following sub-sampling of spike data was performed. For post-weanling animals 30%, and for adult animals 50% of all recorded spikes from each place cell were removed at random from each cell and the stability of place cells across- and within-trials re-analysed with the sub-sampled datasets. Data recorded from pre-weanling animals was not sub-sampled. This procedure had the effect of matching the overall mean firing rates of the post-weanling, and adult, place cells to the mean firing rate of pre-weanling place cells. The random sub-sampling was repeated 100 times, and scores of place cell stability and place cell peak-to-wall distance were calculated for each repeat. The final ANOVA and regressions were performed using the median stability and peak-to-wall distance for each place cell, across the 100 repeated sub-samples.

Equalising firing rates between the edge and centre of the environment. In order to equalise the firing rates of place cells in the edge and centre zones of the environment, the population of place cells was sub-sampled such that the overall mean firing rate of place cells with peaks located in the edge and centre zones of the environment were matched, within each age group (pre-weanling, post-weanling, adult). Overall mean firing rates for zones were matched by the following method: within each age group, the place cells in each zone were sorted by their firing rate, and the highest rate place cells in the higher mean rate zone, and the lowest rate place cells in the lower rate zone, were removed from the dataset until the mean firing rate for the two zones was matched to within 0.01Hz. Place cells were removed from the two zones in proportion to the sample N in each zone (i.e. if there were twice the number of place cells in the edge zone, two cells would be removed from the edge group for every one from the centre group). After data sub-sampling the analyses performed for figure 2 of the main manuscript were run on the population of remaining cells.

Bayesian decoding with soft boundaries. To investigate whether the geometrical constraints on accuracy near the edges, as opposed to at the centre of the environment affected position decoding accuracy, the decoding was run with ‘soft boundaries’, whereby the actual environment was embedded into a larger space, into which position could potentially be decoded. Before estimation of $P(x|\boldsymbol{n})$, the recorded positions of the rat, x, were shifted by 30cm in both the x and y dimensions, while the maps of both position probability, $\mathrm{P}(x)$, and mean firing rate, $f_i(x)$, were padded with a 12 bin (equivalent to 30cm) wide border on all sides, thus creating a ‘soft boundary’ around the actual visited environment. As $P(\boldsymbol{n}|x)$ is defined for the extent of the firing rate map $f_i(x)$, and $\mathrm{P}(x)$ within

the soft boundary was non-zero (always set to the overall mean dwell probability for the trial), the animal's position could, potentially, be decoded to within the soft boundary. After deriving the most likely estimate of $P(x|\boldsymbol{n})$, the difference between the actual and predicted positions was calculated, and accuracy (1/error+1) derived, as for the main analysis, regardless of whether the decoded position was in the actual environment or in the soft boundary. The quadrant mean maps of accuracy (figure S3E, G) depict only the actual environment, and not the soft boundary, as these maps display the mean accuracy for each *visited* spatial bin, and the soft boundary was not, by definition, visited by the rat. Two different versions of the analysis were run, with different definitions of the mean firing rate $f_i(x)$ in the soft boundary. For figure S3E-F, the firing rate for each cell *i,* for the whole soft boundary region, was defined as the grand mean firing rate for the cell *i* in the actual environment. For figure S3G-H, for each cell *i*, the spatial bins of the soft boundary were set to a random selection of values taken from the firing rate map of cell *i* in the actual environment.

Supplemental References

Burnham, K., and Anderson, D. (2002). Model Selection and Multimodel Inference A Practical Information-Theoretic Approach (New York: Springer-Verlag).

Tan, H.M., Bassett, J.P., O'Keefe, J., Cacucci, F., and Wills, T.J. (2015). The Development of the Head Direction System before Eye Opening in the Rat. Curr. Biol. *25*, 479–483.

Wills, T.J., Cacucci, F., Burgess, N., and O'Keefe, J. (2010). Development of the hippocampal cognitive map in preweanling rats. Science (80-.). *328*, 1573–1576.

Zar, J.H. (2010). Biostatistical Analysis (Prentice Hall).
